# Regulation of γ-Aminobutyrate (GABA) Utilization in *Corynebacterium glutamicum* by the PucR-Type Transcriptional Regulator GabR and by Alternative Nitrogen and Carbon Sources

**DOI:** 10.3389/fmicb.2020.544045

**Published:** 2020-10-27

**Authors:** Lingfeng Zhu, Christina Mack, Astrid Wirtz, Angela Kranz, Tino Polen, Meike Baumgart, Michael Bott

**Affiliations:** IBG-1: Biotechnology, Institute of Bio- and Geosciences, Forschungszentrum Jülich, Jülich, Germany

**Keywords:** *Corynebacterium glutamicum*, *Actinobacteria*, γ-aminobutyrate, PucR-type regulator GabR, cAMP-dependent regulator GlxR, nitrogen metabolism, carbon metabolism

## Abstract

γ-Aminobutyric acid (GABA) is a non-proteinogenic amino acid mainly formed by decarboxylation of L-glutamate and is widespread in nature from microorganisms to plants and animals. In this study, we analyzed the regulation of GABA utilization by the Gram-positive soil bacterium *Corynebacterium glutamicum*, which serves as model organism of the phylum *Actinobacteria.* We show that GABA usage is subject to both specific and global regulatory mechanisms. Transcriptomics revealed that the *gabTDP* genes encoding GABA transaminase, succinate semialdehyde dehydrogenase, and GABA permease, respectively, were highly induced in GABA-grown cells compared to glucose-grown cells. Expression of the *gabTDP* genes was dependent on GABA and the PucR-type transcriptional regulator GabR, which is encoded divergently to *gabT*. A Δ*gabR* mutant failed to grow with GABA, but not with glucose. Growth of the mutant on GABA was restored by plasmid-based expression of *gabR* or of *gabTDP*, indicating that no further genes are specifically required for GABA utilization. Purified GabR (calculated mass 55.75 kDa) formed an octamer with an apparent mass of 420 kDa and bound to two inverted repeats in the *gabR*-*gabT* intergenic region. Glucose, gluconate, and *myo*-inositol caused reduced expression of *gabTDP*, presumably via the cAMP-dependent global regulator GlxR, for which a binding site is present downstream of the *gabT* transcriptional start site. *C. glutamicum* was able to grow with GABA as sole carbon and nitrogen source. Ammonium and, to a lesser extent, urea inhibited growth on GABA, whereas L-glutamine stimulated it. Possible mechanisms for these effects are discussed.

## Introduction

γ-Aminobutyric acid (GABA) is a non-proteinogenic amino acid, which is widespread in nature from microorganisms to plants and animals. GABA is formed by many gram-negative and gram-positive bacteria as part of the acid stress response (Cotter and Hill, 2003; Foster, 2004; Krulwich et al., 2011; De Biase and Pennacchietti, 2012). The irreversible decarboxylation of L-glutamate by glutamate decarboxylase results in the formation of GABA and carbon dioxide and consumes a proton within the cytoplasm, thereby acting against acid stress. An antiporter takes up L-glutamate and exports GABA. This acid stress response can also start from L-glutamine, which is first converted to L-glutamate and ammonia by a glutaminase (Pennacchietti et al., 2018). The human gut microbiome contains several GABA-producing or GABA-consuming species, and there might be a connection between GABA production in the gut and depressive disorders (Strandwitz et al., 2019). In plants, GABA plays a role in stress responses and at the interface of carbon and nitrogen metabolism (Michaeli and Fromm, 2015). In the brain of mammals, GABA is an important neurotransmitter (Abdou et al., 2006; Ben-Ari et al., 2007). GABA has also gained much attention as a building block for the synthesis of 2-pyrrolidone and the biodegradable polyamide nylon 4 (Kawasaki et al., 2005). Therefore, microbial synthesis of GABA has frequently been studied (Krulwich et al., 2011; Dhakal et al., 2012; Jorge et al., 2016; Xu et al., 2017).

Due to its widespread occurrence in nature, GABA can be used by many bacteria as carbon and nitrogen source. After uptake by specific transporters (usually named GabP), a transaminase (GabT) converts GABA and 2-oxoglutarate in a pyridoxal 5-phosphate (PLP)-dependent reaction to succinate semialdehyde and glutamate. Succinate semialdehyde is then oxidized by an NAD(P)^+^-dependent succinate semialdehyde dehydrogenase (GabD) to succinate, an intermediate of the central metabolism (Scott and Jakoby, 1958; Dover and Halpern, 1972; Feehily and Karatzas, 2013). Despite the fact that many bacteria possess the characteristic genes involved in GABA metabolism, their regulation has so far only been studied in a few species such as *Bacillus subtilis*, *Bacillus thuringiensis*, or *Escherichia coli*. The best-studied regulator is probably GabR of *B. subtilis* (designated here GabR_Bs_), which belongs to the MocR/GabR subfamily of the GntR family of transcriptional regulators (Edayathumangalam et al., 2013; Wu et al., 2017). GabR_Bs_ consists of an N-terminal helix-turn-helix (HTH) domain and a C-terminal aminotransferase domain and activates its target genes *gabTD* in the presence of GABA and PLP (Belitsky, 2004). Surprisingly, in the close relative *B. thuringiensis*, the regulation of GABA metabolism is different. Here, the regulator GabR_Bt_ belongs to the family of bacterial enhancer binding proteins that function as activators of σ^54^-dependent promoters. GabR_Bt_ is composed of a Per-ARNT-Sim (PAS) domain, a σ^54^ interaction domain, and a C-terminal HTH domain and activates its target gene *gabT* in the presence of GABA and succinate semialdehyde (Zhu et al., 2010; Peng et al., 2014). In *E. coli*, the genes *gabTDP* are presumably organized in an operon *gabTDPC* together with *gabC*, and activated under nitrogen limitation by the Nac regulator (Schneider et al., 2002). The product of *gabC* is a transcriptional regulator belonging to the FadR subfamily of the GntR family of transcriptional regulators and seems to repress this operon. However, the exact physiological function of GabC_Ec_ is unclear up to now (Schneider et al., 2002). GABA is also an intermediate of putrescine degradation to succinate in *E. coli*, which involves PuuE as isoenzyme of GabT and PuuC as isoenzyme of GabD (Kurihara et al., 2010; Schneider and Reitzer, 2012). In summary, the regulation of genes involved in GABA metabolism appears to be rather diverse in bacteria.

**FIGURE 1:**
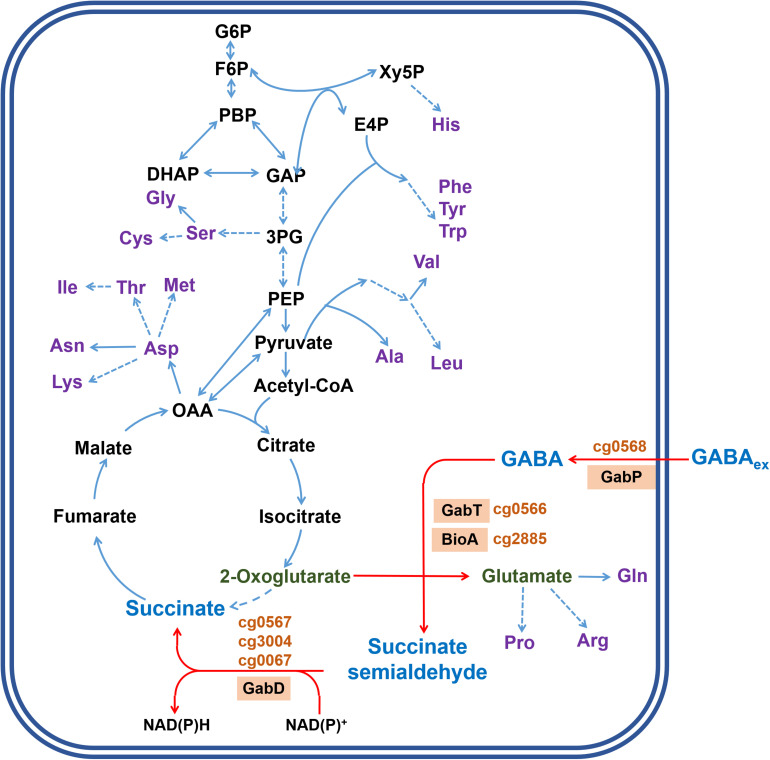
Model of GABA metabolism in *C. glutamicum* and integration into central metabolism. The metabolites and reactions specifically involved in GABA metabolism are indicated by blue letters and red arrows, respectively. The proteins involved in GABA transport and degradation and the corresponding locus tags are indicated in orange. GabP is a sodium ion-coupled secondary transporter for GABA uptake (Zhao et al., 2012). GabT functions as γ-aminobutyrate aminotransferase (Shi et al., 2017). BioA (cg2885) was reported to also possess some GABA aminotransferase activity (Shi et al., 2017). GabD (cg0567) as well as two further proteins encoded by cg0067 and cg3004 are annotated as succinate semialdehyde dehydrogenases (Kalinowski et al., 2003). GABA_ex_ represents extracellular GABA. Metabolites involved in central metabolism are shown in black letters, amino acids in purple letters. Solid blue arrows indicate conversions catalyzed by a single enzyme, dashed lines represents conversions composed of several enzymatic steps. Abbreviations: DHAP, dihydroxyacetone phosphate; E4P, erythrose 4-phosphate; F6P, fructose 6-phosphate; FBP, fructose 1,6-bisphosphate; GAP, glyceraldehyde 3-phosphate; G6P, glucose 6-phosphate; OAA, oxaloacetate; PEP, phosphoenolpyruvate; 3PG, 3-phosphoglycerate; Xy5P, xylulose 5-phosphate.

*Corynebacterium glutamicum* is a gram-positive soil bacterium widely used for the industrial production of amino acids, in particular the flavor enhancer L-glutamate and the feed additive L-lysine (Eggeling and Bott, 2005). The wild type is unable to synthesize GABA, but recombinant strains for GABA production were constructed (Shi and Li, 2011; Takahashi et al., 2012; Shi et al., 2013; Jorge et al., 2016). In the course of these studies, it became clear that *C. glutamicum* is able to degrade GABA and a number of proteins involved in this process were studied (Figure 1). GabP (Cg0568) is a secondary Na^+^-dependent transporter for GABA uptake with a *K*_*m*_ value of 34.2 ± 1.1 μM and a *V*_*max*_ of 67.3 ± 1.0 nmol min^–1^ (mg cell dry weight)^–1^ at pH 7.5 and essential for GABA utilization (Zhao et al., 2012). GabT (Cg0566) is a GABA transaminase and the purified enzyme has a specific activity of 1.34 U/mg protein and *K*_*m*_ values of 10.65 mM for GABA and 9.21 mM for 2-oxoglutarate (Shi et al., 2017). GabT was shown to be a tetramer and the crystal structure in complex with PLP-GABA allowed the identification of the key residues that contribute to the formation of the active site (Hong and Kim, 2019). Since a *gabT* deletion strain was still able to degrade GABA, the presence of another transaminase catalyzing this reaction was suggested (Ni et al., 2015). The protein encoded by *bioA* (cg2885) was recently reported to possess GABA transaminase activity, but the specific activity of the purified BioA enzyme was only 2% of the GabT activity (Shi et al., 2017). With respect to succinate semialdehyde dehydrogenase, three genes were annotated to encode this enzyme, namely *gabD* (cg0566), cg0067, and cg3004 (Figure 1) (Kalinowski et al., 2003). None of the corresponding proteins has been biochemically characterized to our knowledge.

In this study, our aim was to investigate the regulation of GABA utilization in *C. glutamicum*. We show that the *gabTDP* operon is regulated by a specific transcriptional regulator, GabR, which does not belong to the regulator families described above for GabR proteins of other bacteria, but to the PucR family, further confirming the heterogeneity in transcriptional regulation of the genes involved in GABA transport and metabolism. GabR presumably requires GABA for transcriptional activation, but not for DNA-binding. The *gabTDP* operon is probably also regulated by the cAMP-dependent global regulator GlxR, which is proposed to repress expression when alternative carbon sources are present. Besides alternative carbon sources, also alternative nitrogen sources have a profound influence on GABA utilization, with ammonium and urea acting as inhibitors and glutamine serving as stimulator.

## Materials and Methods

### Bacterial Strains, Plasmids and Growth Conditions

The bacterial strains and plasmids used in this study are listed in Table 1. The *C. glutamicum* ATCC 13032 type strain served as wild type (WT). *C. glutamicum* was routinely cultivated at 30°C. For precultivation of *C. glutamicum*, brain heart infusion (BHI) medium (Becton Dickinson GmbH, Heidelberg, Germany) with 90 g L^–1^ sorbitol was used. The cells of these precultures were harvested by centrifugation (5,000 *g*, 4°C, 10 min), and washed twice with phosphate buffer (100 mM KH_2_PO_4_/Na_2_HPO_4_ pH 7.0). Growth experiments were routinely performed in a BioLector microcultivation system (m2p-labs, Baesweiler, Germany) using 48-well FlowerPlates (m2p-labs) containing 750 μL CGXII minimal medium (Keilhauer et al., 1993) supplemented with 30 mg L^–1^ protocatechuic acid as iron chelator. In the BioLector, growth of the cells is measured as scattered light at 620 nm (Kensy et al., 2009a,b). Please note that the absolute backscatter values of the graphs in this manuscript differ because of the use of different instruments. Due to this, reference cultures were always included to allow comparison between different experiments. For determination of GABA and glucose consumption, cells were cultured in 500-mL baffled shake flasks with 50 mL medium that were incubated at 30°C and 130 rpm. *E. coli* was grown at 37°C in lysogeny broth (Bertani, 1951). When required for plasmid maintenance, media were supplemented with kanamycin (50 μg mL^–1^ for *E. coli*, 25 μg mL^–1^ for *C. glutamicum*) or chloramphenicol (20 μg mL^–1^).

**TABLE 1:** Bacterial strains and plasmids used in this study.

Strain or plasmid	Relevant characteristics	Source or references
***E. coli***		
DH5α	F^–^ Φ80*dlac*Δ(*lacZ*)M15 Δ(*lacZYA-argF*) U169 *endA1 recA1 hsdR17* (r_K_^–^, m_K_^+^) *deoR thi-1 phoA supE44*λ^–^ *gyrA96 relA1*; strain used for cloning procedures	(Hanahan, 1983)
BL21(DE3)	F- *ompT hsdS*_B_ (r_B_-, m_B_-) *gal dcm* (DE3); host for protein production	(Studier and Moffatt, 1986)
***C. glutamicum***		
ATCC 13032 (Cg WT)	Biotin-auxotrophic wild type	(Kinoshita et al., 1957)
Δ*gabR*	WT derivative with an in-frame deletion of *gabR* (cg0565)	This work
Δ*gdh* (LNΔGDH)	ATCC 13032 with deletion of *gdh* (cg2280)	(Müller et al., 2006)
Δ*glnA* (LNΔGS)	ATCC 13032 with deletion of *glnA* (cg2429)	(Müller et al., 2006)
Δ*gltBD* (LNΔ*gltBD*)	ATCC 13032 with deletion of *gltB* (cg0229) and *gltD* (cg0230)	(Rehm et al., 2010)
Δ*glnA*Δ*glnA2*Δ*gdh* (DA-2)	ATCC 13032 with deletion of *gdh* (cg2280), *glnA* (cg2429) and *glnA*2 (cg2477)	(Walter et al., 2008)
**Plasmids**		
pK19*mobsacB*	Kan^R^; plasmid for allelic exchange in *C. glutamicum*; (pK18 *ori*V*_*E.c*_*., *sacB*, *lacZ*α)	(Schäfer et al., 1994)
pK19*mobsacB*-Δ*gabR*	Kan^R^.; pK19*mobsacB* derivative containing a PCR product covering the up- and downstream regions of *gabR* (cg0565)	This work
pAN6	Kan^R^; *C. glutamicum*/*E. coli* shuttle vector for regulated gene expression using the P*_*tac*_* promoter, derivative of pEKEx2.	(Frunzke et al., 2008)
pAN6-*gabR*	Kan^R^; pAN6 derivative for expression of *gabR* (cg0565) under control of P*_*tac*_*	This work
pAN6-*gabTDP*	Kan^R^; pAN6 derivative for expression of *gabTDP* cluster	This work
pET-TEV	Kan^R^; pET28b derivative for overexpression of genes in *E. coli*, adding an N-terminal decahistdine tag and a TEV protease cleavage site to the target protein (pBR322 oriV_*E.c.*_, P_T7_, *lacI*)	(Bussmann et al., 2010)
pET-TEV-*gabR*	Kan^R^; pET-TEV derivative for overproduction of GabR (cg0565)	This work
pJC1-P*_*tac*_*-*eyfp*	Kan^R^; pJC1 derivative containing the *eyfp* gene under the control of P*_*tac*_*	(Kortmann et al., 2015)
pJC1-venus-term	Kan^R^; *E. coli*-*C. glutamicum* shuttle vector, pJC1 derivative carrying the venus coding sequence and additional terminators	(Baumgart et al., 2013)
pJC1-P*_*gabT*_*-eYFP	Kan^R^; pJC1-venus-term derivative carrying the promoter of *gabT* (cg0566) fused to *eyfp*	This work
pJM0462	Cm^R^; pASK-IBA-3C derivative with the coding sequence of *gabT* (cg0566, NCgl0462)	(Marienhagen et al., 2005)

### Recombinant DNA Work and Construction of Deletion Mutants

Routine methods such as PCR, DNA restriction and ligation were performed using standard protocols (Sambrook et al., 1989; van der Rest et al., 1999). All oligonucleotides used in this study are listed in Supplementary Table 1 and were synthesized by Eurofins Genomics (Ebersberg, Germany). The enzymes for recombinant DNA work were obtained from New England Biolabs (Frankfurt, Germany) and Thermo Fisher Scientific (Vilnius, Lithuania). The correctness of the insert sequences of all recombinant plasmids was verified by DNA sequencing (Eurofins Genomics, Ebersberg, Germany).

The *C. glutamicum* Δ*gabR* strain was constructed by allelic exchange using the plasmid pK19*mobsacB* (Schäfer et al., 1994) as described before (Niebisch and Bott, 2001). For construction of pK19*mobsacB*-Δ*gabR*, the oligonucleotide pairs cg0565frontF/R and cg0565backF/R were used to amplify DNA regions of approximately 500 bp upstream and downstream of *gabR* using *C. glutamicum* WT genomic DNA as template. The resulting PCR products were fused by Gibson assembly (Gibson et al., 2009) with pK19*mobsacB* which had been digested with *Bam*HI and *Pst*I. After successful construction, pK19*mobsacB*-Δ*gabR* was used to transform competent *C. glutamicum* WT. The transformed cells were first selected for kanamycin resistance and subsequently for tolerance toward sucrose. Successful deletion of *gabR* was confirmed by colony PCR with the oligonucleotides cg0565checkF and cg0565checkR, which anneal outside of the deleted regions. Out of six analyzed clones, three showed the desired *gabR* deletion, while three clones represented the WT situation.

For construction of plasmid pAN6-*gabR*, the *gabR* coding region was amplified by PCR using the oligonucleotides 0565F and 0565R and chromosomal DNA of *C. glutamicum* WT as template. After digestion with *Nde*I and *Nhe*I, the PCR product was cloned into the expression plasmid pAN6 cut with the same restriction enzymes using the Rapid DNA Ligation Kit (Roche Diagnostics, Mannheim, Germany). The plasmid pET-TEV-*gabR* was constructed similar to pAN6-*gabR*. The *gabR* gene was amplified with the oligonucleotides 0565F and petfhis0565R, the PCR product was digested with *Nde*I and *Hin*dIII and ligated with pET-TEV cut with the same restriction enzymes. For construction of plasmid pAN6-*gabTDP*, the genes *gabTDP*, were amplified by PCR using the oligonucleotide pair gabtdpF/gabtdpR and chromosomal DNA of *C. glutamicum* WT as template. The resulting PCR product was fused with pAN6 cut with *Nde*I and *Nhe*I by Gibson assembly.

For construction of plasmid pJC1-P*_*gabT*_*-eYFP, a 500-bp fragment covering the *gabTDP* promoter region immediately upstream of the *gabT* start codon was amplified using the oligonucleotide pair PgabTF/PgabTR with genomic DNA of *C. glutamicum* WT as template. The eYFP-encoding gene was amplified with the oligonucleotide pair eYFPF/eYFP-termR and plasmid DNA of pJC1-P*_*tac*_*-*eyfp* as template. The two PCR fragments were fused by Gibson assembly with pJC1-venus-term, which had been digested with *Bam*HI and *Spe*I.

### DNA Microarray Analysis

Comparative transcriptome analysis using DNA microarrays was performed as described previously (Vogt et al., 2014), except that (NH_4_)_2_SO_4_ was omitted from the medium to exclude ammonium and 20 mM K_2_SO_4_ was added to avoid a possible sulfate limitation. The medium still contains 83 mM urea as nitrogen source. *C. glutamicum* WT was cultivated in this modified medium with either 62.5 mM GABA or with 41.7 mM glucose as carbon source, corresponding to 250 mM C. When an optical density at 600 nm (OD_600_) of 5 was reached [measured with an Ultrospec 2100pro spectrophotometer (Biochrom, Berlin, Germany)], the cells were harvested by centrifugation (4000*g*, 10 min, 4°C). The cell pellet was subsequently frozen in liquid nitrogen and stored at −80°C. Total RNA isolation of *C. glutamicum* cells was performed using the RNeasy Mini kit (Qiagen, Hilden, Germany), and the RNA was kept at −80°C until further use. Fluorescently labeled cDNA copies of total RNA of *C. glutamicum* were prepared using SuperScript III reverse transcriptase (Life Technologies, Darmstadt, Germany). To remove unincorporated fluorophores, the probes were purified using Amicon Centrifugal Filters (Merck Millipore, Darmstadt, Germany). The cDNA probes labeled with Cy3 and Cy5 were hybridized using Agilent’s Gene Expression Hybridization Kit (Waldbronn, Germany), hybridization oven and hybridization chamber. After 16 h of hybridization at 65°C, the microarrays were washed using Agilent’s Wash Buffer Kit according to the manufacturer’s instructions. Signal acquisition was performed with a GenePix 4000B laser scanner and GenePix Pro 7.0 software (Molecular Devices, Sunnyvale, CA, United States). For background correction of spot intensities, ratio calculation and ratio normalization, data were processed using the BioConductor R-packages limma and marray^1^. The full microarray data sets of this study have been deposited in the NCBI Gene Expression Omnibus and can be found under the GEO accession number GSE138829.

### Determination of the Transcriptional Start Sites (TSSs)

The TSS of *gabT* was initially determined using the 5′/3′ RACE Kit (Roche Diagnostics, Mannheim, Germany). In brief, RNA was extracted from *C. glutamicum* WT cells cultivated with GABA as sole carbon and nitrogen source using an RNeasy Mini Kit (Qiagen). Then transcription of specific mRNA sequences into first-strand cDNA was performed using the extracted RNA and oligonucleotide GABTTS1R (Supplementary Table 1). A homopolymeric A-tail was added to the 3′-end of the first-strand cDNA using recombinant terminal transferase and dATP. Finally, the amplification of dA-tailed cDNA was performed using first and second round PCR using the primers GABTTS2R and GABTTS3R, respectively (Supplementary Table 1). The PCR products were sequenced to determine the TSS. The TSS determination was performed independently for three biological replicates.

In an alternative approach, the TSSs present within the *gabR-gabTDP* region were analyzed by RNAseq using RNA of WT cells cultivated with GABA as sole carbon and energy source. Three independent cultures (5 ml BHI) were inoculated with a single colony from a fresh agar plate and cultivated at 30°C and 170 rpm during the day. The cells were washed two times with 0.9% (w/v) NaCl and used to inoculate the second precultures of 20 ml CGXII medium with 2% (w/v) glucose in a 1:100 dilution. These cultures were cultivated at 30°C and 130 rpm overnight. For the main cultures, 50 ml CGXII medium without ammonium and urea but with 62.5 mM GABA as carbon and nitrogen source were inoculated with the precultures to an OD_600_ of 1. The main cultures were incubated at 30°C and 130 rpm and harvested after 6 h at an OD_600_ of 2.5-2.7. RNA isolation was performed with the RNeasy Mini Kit (Qiagen, Hilden, Germany). For the sequencing, RNA of all three cultures was mixed in equal amounts. Further RNA processing and sequencing was performed by Vertis Biotechnologie AG (Freising-Weihenstephan, Germany). Processing and mapping of the 10,117,524 single-end sequenced reads was performed with the CLC Genomics Workbench (Qiagen Aarhus A/S). Reads were trimmed by removing adapter sequences using the Trim Sequences tool and filtered for Phred quality scores < 30 (Ewing and Green, 1998). Trimmed reads (10,052,707) were mapped to the *C. glutamicum* reference sequence (BX927147). Automatic detection of TSSs was done with ReadXplorer (Hilker et al., 2014) using the following parameters: (i) Only single perfect mappings without errors were considered. (ii) Minimum percentage of coverage increase was set to 100% and minimum number of reads starts to 20. Every possible TSS for cg0565-cg0568 was manually checked including TSS with less than 20 reads.

### Protein Production and Purification

For GabR production and purification, *E. coli* BL21(DE3) harboring pET-TEV-*gabR* was first cultivated in 5 mL LB medium with 50 μg mL^–1^ kanamycin in 20 mL glass tubes at 37°C with shaking overnight. Subsequently, 2 mL preculture was used to inoculate 200 mL LB medium with kanamycin in a 500 mL shake flask, which was cultivated at 37°C and 120 rpm. When the culture reached an OD_600_ of 0.6, 0.2 mM isopropyl β-D-1-thiogalactopyranoside (IPTG) was added to induce *gabR* expression. After induction, the cells were further cultivated at 16°C for 8 h and harvested by centrifugation (4,000*g*, 10 min, 4°C). The harvested cells were suspended in binding buffer (20 mM Tris–HCl, 500 mM NaCl, 1 mM DTT, 10 mM imidazole, pH 7.8) and disrupted by sonication for 20 minutes while cooling on ice with a UP 200 S ultrasonic device (Hielscher, Germany) using a power amplitude of 55% and a pulse cycle of 0.3. The resulting cell extract was centrifuged at 12,000*g* for 20 min to remove cell debris and the supernatant was subjected to Ni^2+^ affinity chromatography using Ni-NTA Superflow (Qiagen, Hilden, Germany). The column was equilibrated with binding buffer. After adding the cell-free extract, the column was washed first with three column volumes of binding buffer and then with three column volumes of wash buffer (20 mM Tris–HCl, 500 mM NaCl, 1 mM DTT, 80 mM imidazole, pH 7.8). GabR protein with an N-terminal decahistidine tag was eluted with elution buffer (20 mM Tris–HCl, 500 mM NaCl, 1 mM DTT, and 300 mM imidazole, pH 7.8). Subsequently, the His-tag was cleaved off with tobacco etch virus (TEV) protease (Kapust and Waugh, 1999) by incubation of 12 mg His-tagged GabR with 0.24 mg His-tagged TEV protease overnight at 4°C in TEV buffer (25 mM Tris–HCl, pH 8.0, 0.25 mM EDTA, 1 mM DTT). Subsequently, GabR was further purified by gel filtration with a Superdex^TM^ 200 increase 10/300 GL column (GE Healthcare, Freiburg, Germany) connected to an Äkta^TM^ Pure25 system (GE Healthcare) using 100 mM phosphate buffer pH 7.0 with 1 mM DTT and a flow rate of 0.75 mL min^–1^. The elution volumes of the standard proteins were 9.41 mL for thyroglobulin (669 kDa), 10.87 mL for apoferritin (443 kDa), 12.06 mL for amylase (200 kDa), 12.85 mL for alcohol dehydrogenase (150 kDa), 14.27 ml for BSA (66 kDa), 16.26 mL for carbonic anhydrase (29 kDa), and 17.34 mL for cytochrome *c* (12.4 kDa).

Overproduction and purification of the transaminase GabT were performed according to a previous publication (Marienhagen et al., 2005). *E. coli* BL21(DE3) carrying plasmid pJM0462 was precultivated in 5 mL LB medium with 20 μg mL^–1^ chloramphenicol in 20 mL tubes at 37°C and 120 rpm overnight. Subsequently, 2 mL preculture was used to inoculate 200 mL LB medium with 20 μg mL^–1^ chloramphenicol in a 500 mL shake flask, which was incubated at 37°C and 120 rpm. Induction of target gene expression was triggered by addition of 20 μL anhydrotetracycline (2 mg (mL ethanol) ^–1^) when the cultures had reached an OD_600_ of 0.4 to 0.6 and the cultures were further incubated for 8 h at 16°C. Crude extracts were obtained by sonication while cooling on ice. After removal of the cellular debris by centrifugation (15 min, 16,000*g*, 4°C), protein purification was performed by affinity chromatography on ice using Strep-TactinXT Sepharose (IBA, Göttingen, Germany). Buffer W (100 mM Tris–HCl pH 8.0, 150 mM NaCl, 1 mM EDTA) was used for equilibration and washing, and buffer BXT (buffer W containing 50 mM biotin) was used for elution. The purified proteins were analyzed by SDS-PAGE. Protein concentrations were determined using the BC Protein Assay Kit (Interchim Uptima, Montlucon Cedex, France) with bovine serum albumin (BSA) as standard.

### Electrophoretic Mobility Shift Assays (EMSAs)

Electrophoretic mobility shift assays were performed as described previously (Wennerhold and Bott, 2006). The DNA fragments (100 ng, 30 – 500 bp) were incubated with purified GabR protein (0 – 2.6 μM monomer) in binding buffer (10 mM Tris–HCl pH 7.5, 50 mM NaCl, 5% (v/v) glycerol, 0.005% (v/v) Triton X-100) for about 20 min at room temperature. Electrophoresis was performed using 6% or 8% (w/v) native polyacrylamide gels in an ice bath with TB buffer (89 mM Tris–HCl pH 8.2, 89 mM boric acid) as running buffer. A pre-run without samples was carried out, so that buffer differences between the gel and the running buffer were adjusted (180 V, 1.5 h). After the pre-run, the samples were mixed with sample buffer (0.01% (w/v) xylene cyanol, 0.01% (w/v) bromophenol blue, 20% (v/v) glycerol, in 1 × TB buffer) and loaded onto the gel. Electrophoresis was performed with the same conditions as the pre-run for 40 minutes and the gels were subsequently stained with SYBR green (Sigma-Aldrich, Darmstadt, Germany).

### HPLC Analysis of GABA and Glucose

For the determination of GABA and glucose in culture superntants, 1-mL culture aliquots were taken at selected time points, centrifuged for 5 min at 13,000*g*, and the supernatant was stored at −20°C. Thawed samples were filtered (0.2 μm syringe filter, Whatman^TM^, GE Healthcare, Freiburg, Germany) prior to HPLC analysis. The GABA concentration was quantified as *ortho*-phthalaldehyde derivative by reverse phase chromatography using an Agilent 1290 Infinity I LC system (Agilent, Santa Clara, CA, United States) equipped with a 5-micron 4.6 × 12.5 mm protective column and a Zorbax Eclipse AAA 3.5 micron 4.6 × 75 mm separating column. A gradient of sodium borate buffer (A: 10 mM Na_2_HPO_4_, 10 mM Na_2_B_4_O_7_, pH 8.2) and methanol (B) was used as eluent. Starting initially with 100% A, B was increased within 9.8 min from 0% to 57%. At 10 min, B was set to 100% and hold for 2.5 min. Re-equilibration to 100% A was started after 12.5 min and terminated after 14 min. The *ortho*-phthalaldehyde derivative was detected by a fluorescence detector using an excitation wavelength of 340 nm and an emission wavelength of 450 nm. The flow rate was 2 mL/min and the temperature was kept constant at 40°C.

Glucose was quantified essentially as described (Richhardt et al., 2012) using an Agilent LC-1100 system (Agilent, Santa Clara, CA, United States) equipped with a Carbo-Ca Guard Cartridge (Phenomenex, Aschaffenburg, Germany) and a Rezex^TM^ RCM-Monosaccharide 300 × 7.8 mm column (Phenomenex, Aschaffenburg, Germany). Separation was performed at 80°C with water as eluent at a flow rate of 0.6 mL/min. Glucose was detected with a refraction index detector (35°C). Glucose was calibrated in a range of 0.1 to 8 g/L, with a retention time of 11.2 min.

### Assay for GABA Transaminase Activity

The activity of GABA transaminase was measured as described (Marienhagen et al., 2005). Briefly, the assay buffer contained 200 mM Tris–HCl pH 8, 0.25 mM pyridoxal 5′-phosphate, 20 mM GABA, and 20 mM 2-oxoglutaric acid. To test if ammonium affects GABA transaminase activity, 30 mM (NH_4_)_2_SO_4_ was added. The reaction mixture (initial volume 10 ml) was preincubated for 2 min at 30°C and started by the addition of purified protein at a final concentration of 3 μg mL^–1^. Six 500 μl samples were collected over a period of 26 min and the reaction was immediately terminated by mixing each sample with 300 μL of 5% (v/v) perchloric acid and 38% (v/v) ethanol. After this, the sample was neutralized by addition of 200 μL of 20 mM Tris–HCl (pH 8) with 23 mM K_2_CO_3_. The precipitated salts were removed by centrifugation (10 min, 16,000*g*). Subsequently, the glutamate concentration in the sample was measured by HPLC (Agilent 1260 series) equipped with an Agilent Eclipse XDB-C18 column using a variable wavelength detector and a fluorescence detector. Elution was performed with a mixture of 43% (v/v) buffer A [10 mM Na_2_HPO_4_, 10 mM Na_2_B_4_O_2_ (pH 8.2)] and 57% (v/v) methanol at a flow rate of 2 mL/min for 14 minutes. Before chromatographic separation, amino acids were derivatized with *o*-phthaldialdehyde (Lindroth and Mopper, 1979). 25–1000 μM sodium glutamate was used as standard.

## Results

### Transcriptome Comparison of WT Cells Grown With GABA or Glucose

**FIGURE 2:**
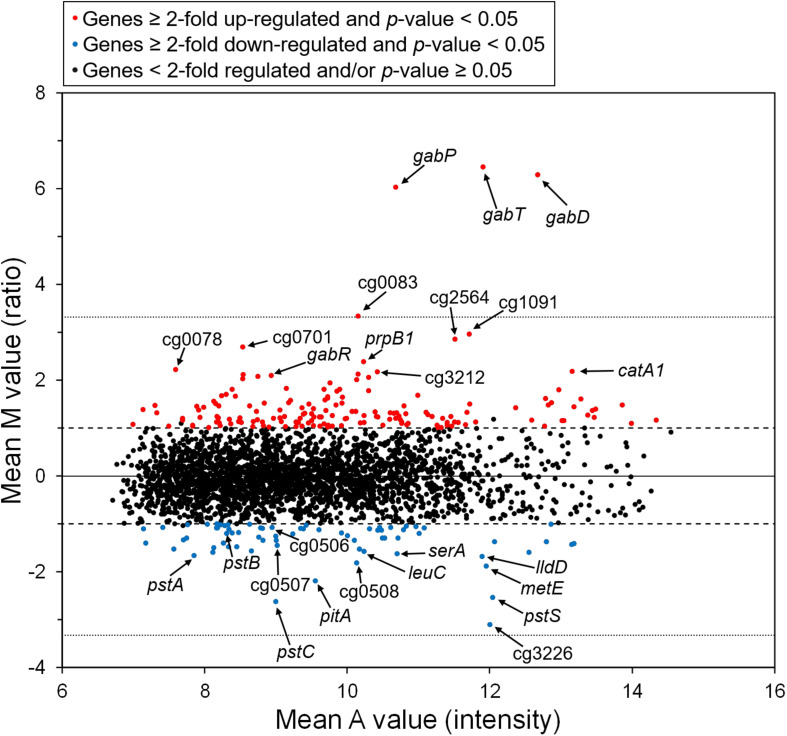
M/A plot showing differential gene expression in *C. glutamicum* WT cells grown with GABA and urea compared to WT cells grown with glucose and urea. The data shown are based on four two-channel DNA microarray hybridizations starting with cDNA from four independent biological replicates. The dashed lines indicate a 2-fold altered mRNA ratio, the dotted lines a 10-fold altered mRNA ratio.

To analyze the influence of GABA on global gene expression, we compared the transcriptomes of cells cultivated in modified CGXII minimal medium lacking (NH_4_)_2_SO_4_ and containing either GABA or glucose as carbon source. In GABA-grown cells, 163 genes showed a ≥ 2-fold increased mRNA level and 71 genes a ≥ 2-fold lowered mRNA level compared to glucose-grown cells (Supplementary Table 2). The genes showing the by far strongest upregulation in GABA-grown cells were *gabTDP* (87-, 78-, and 65-fold, respectively) (Figure 2 and Supplementary Table 2). Interestingly, also expression of the *gabR* gene located upstream and divergent to *gabTDP* was four-fold increased in GABA-grown cells. The *gabR* gene encodes a transriptional regulator (see below). Among the other genes upregulated in GABA-grown cells, only cg0083 encoding a putative nicotinamide mononucleotide transporter showed a more than 10-fold increased expression. In general, the upregulated genes belonged to a large variety of functional categories and the same holds true for the genes downregulated in GABA-cultivated cells. The latter group included at least 30 genes for proteins involved in transport, including those for phosphate uptake (*pitA, pstSCAB*), genes involved in amino acid biosynthesis (*leuCD, serA, metE, argC, aroG, glnA*), and genes involved in lactate metabolism (cg3226, *lldD*). In summary, this experiment demonstrated that the *gabTDP* genes were strongly induced in the presence of GABA, indicating that they are subject to transcriptional control. All other differentially expressed genes were significantly less changed under this condition.

### Genomic Location and Conservation of the *gabR-gabTDP* Gene Cluster

**FIGURE 3:**
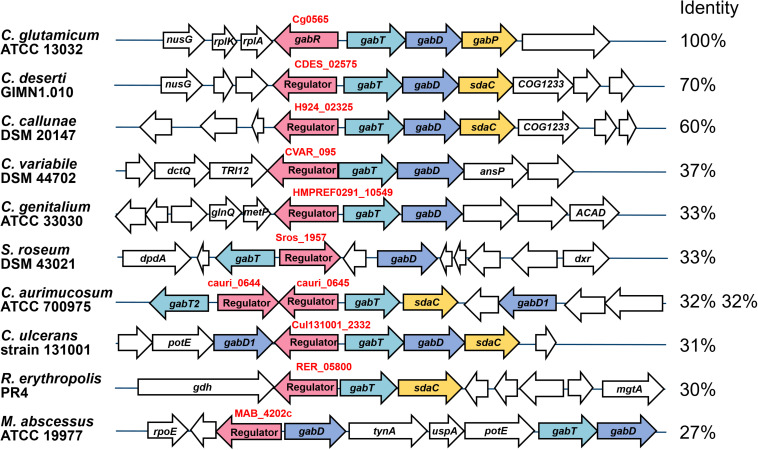
Genomic organization of the *gabR*-*gabTDP* gene cluster in different *actinobacterial* species. Conserved genes are marked with different colors: *gabT* (γ-aminobutyrate aminotransferase, light blue), *gabD* (succinate semialdehyde dehydrogenase, blue), *gabP* or *sdaC* (GABA-specific importer, yellow), *gabR* (transcriptional regulator, red). Non-conserved genes located next to the *gabR-gabTDP* cluster are shown colorless. The amino acid sequence identity of GabR homologs with GabR of *C. glutamicum*, which was calculated by MultiGeneBlast (Medema et al., 2013), is given on the right. For *C. aurimucosum*, identity for both GabR homologs is given. The sequences were derived from the following genomes: *Corynebacterium deserti* GIMN1.010 (NZ_CP009220), *Corynebacterium callunae* DSM 20147 (NC_020506), *Corynebacterium variabile* DSM 44702 (NC_015859), *Corynebacterium genitalium* ATCC 33030 (NZ_CM000961), *Streptosporangium roseum* DSM 43021 (NC_013595), *Corynebacterium aurimucosum* ATCC 700975 (NC_012590), *Corynebacterium ulcerans* strain 131001 (NZ_CP010818), *Rhodococcus erythropolis* PR4 (NC_012490) and *Mycobacterium abscessus* ATCC 19977 (CU458896). The figure was generated using MultiGeneBlast (Medema et al., 2013).

The *gabR* gene is located upstream and divergent to *gabTDP* and encodes a transcriptional regulator, which might regulate these genes. To check whether this genomic organization is conserved in related species, which is a hint for a regulatory relationship between these genes, we analyzed the presence and organization of the *gabR-gabTDP* gene cluster in other organisms. As shown in Figure 3, similar gene clusters were found in a number of *Corynebacterium* species and, in a different organization, also in some *Rhodococcus* and *Mycobacterium* species. Interestingly, additional copies of *gabR* and *gabT* are present immediately adjacent to the *gabR-gabTDP* gene cluster in *Corynebacterium aurimucosum*. GabR and its homologs from *Actinobacteria* belong to the PucR protein family (Pfam PF07905) and have a C-terminal DNA-binding HTH motif. The best characterized representative of this family is PucR of *B. subtilis*, which is involved in the regulation of purine catabolism (Schultz et al., 2001; Beier et al., 2002).

### Relevance of *gabR* and the *gabTDP* Operon for Growth With GABA

**FIGURE 4:**
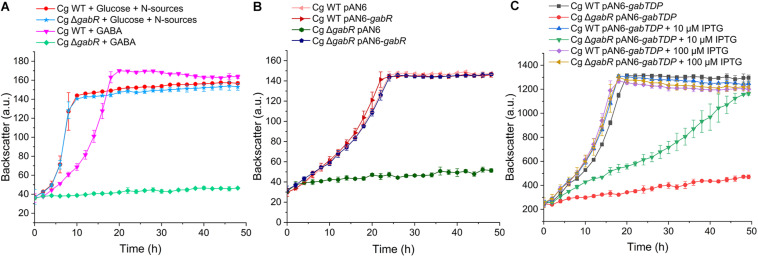
Growth studies with *C. gutamicum* WT and the Δ*gabR* mutant with or without the plasmids pAN6, pAN6-*gabR*, or pAN6-*gabTDP*. All strains were precultivated in BHIS medium and washed with phosphate buffer before inoculation of the main culture. Growth experiments were performed in a BioLector microcultivation system using 48-well Flower plates containing 750 μl CGXII minimal medium with 62.5 mM GABA as sole carbon and nitrogen source or with 41.7 mM glucose as carbon source and 151 mM (NH_4_)_2_SO_4_ and 83 mM urea as nitrogen sources. **(A)** Growth of the *C. glutamicum* Δ*gabR* mutant compared to the WT with glucose or GABA as carbon sources. **(B)** Influence of plasmids pAN6 and pAN6-*gabR* on growth of *C. glutamicum* WT and the Δ*gabR* mutant with GABA as sole carbon and nitrogen source. **(C)** Influence of plasmids pAN6 and pAN6-*gabTDP* on growth of *C. glutamicum* WT and the Δ*gabR* mutant with GABA as sole carbon and nitrogen source. Where indicated, 10 μM or 100 μM IPTG was added to the medium for induction of the P*_*tac*_* promoter controlling expression of *gabTDP*. Since the promoter of pAN6 is known to be slightly leaky, basal transcription of the target genes is independent of IPTG addition. Mean values and standard deviations of three biological replicates are shown.

To investigate whether GabR is involved in the regulation of GABA metabolism, we generated the deletion mutant *C. glutamicum* Δ*gabR* and compared it with the WT. Both strains grew identically with glucose, but the Δ*gabR* mutant had lost the ability to grow with GABA as sole carbon and nitrogen source (Figure 4A). The growth defect of the Δ*gabR* strain with GABA was abolished after transformation with the expression plasmid pAN6-*gabR* harboring *gabR* under control of a leaky *tac* promoter (Figure 4B), which confirmed that the loss of GabR was responsible for the observed phenotype. Whereas full complementation was achieved by basal *gabR* expression in the absence of IPTG, induction of plasmid-borne *gabR* expression with 50 μM IPTG had a negative effect on growth of the Δ*gabR* mutant and the WT in comparison to the non-induced cultures (Supplementary Figure 1). This might be a consequence of too strong expression of the *gabTDP* operon causing e.g., membrane-stress by overexpression of the permease-encoding gene *gabP* or metabolic disturbances by an excessive GABA metabolism.

In a further experiment, we tested whether growth of the Δ*gabR* strain on GABA can be restored by plasmid-based expression of *gabTDP. C. glutamicum* WT and the Δ*gabR* mutant were transformed with pAN6-*gabTDP* and cultivated with GABA as sole carbon and nitrogen source and different IPTG concentrations (Figure 4C). Without IPTG addition, the Δ*gabR* strain with pAN6-*gabTDP* grew very slowly. Addition of 10 μM IPTG significantly improved the growth of this strain and with 100 μM IPTG it grew comparably to the WT carrying pAN6-*gabTDP*. These results suggest that *gabTDP* are the only genes required for growth with GABA that are controlled by GabR.

### Purification of GabR and Determination of the Native Size

**FIGURE 5:**
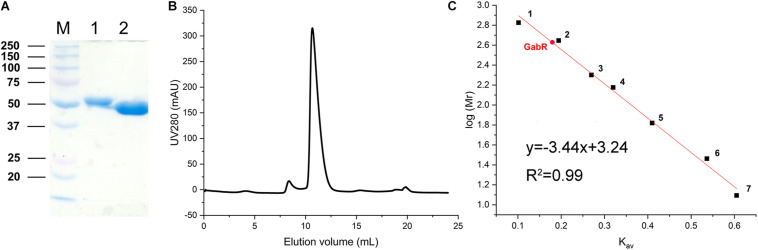
Purification of GabR and determination of its native molecular mass. **(A)** Coomassie-stained SDS-polyacrylamide gel showing marker proteins (M, molecular mass in kDa) and GabR after Ni-NTA affinity chromatography (lane 1) and after His-tag removal with TEV protease (lane 2). **(B)** Size exclusion chromatography of tag-free GabR using a Superdex 200 Increase 10-300GL column (GE Healthcare). Protein was detected by absorbance at 280 nm. **(C)** Calibration curve for the Superdex column obtained with standard proteins: 1, thyroglobulin (669 kDa); 2, apoferritin (443 kDa); 3, amylase (200 kDa); 4, alcohol dehydrogenase (150 kDa); 5, bovine serum albumin (66 kDa); 6, carbonic anhydrase (29 kDa); 7, cytochrome *c* (12.4 kDa). The K_av_ value determined for GabR is marked with a red dot.

The growth experiments with the Δ*gabR* mutant showed that GabR activates *gabTDP* expression. To get further insights into the regulatory mechanism, we purified GabR for interaction studies with the P*_*gabTDP*_* promoter by EMSAs. GabR was overproduced in *E. coli* BL21(DE3) and purified by means of an N-terminal decahistidine tag and Ni-NTA affinity chromatography (Figure 5A). The tag was cleaved off with TEV protease followed by size exclusion chromatography to further purify GabR and determine its native size and oligomeric state (Figure 5B). The peak of GabR appeared at an elution volume of 10.67 mL. Based on the calibration curve (K_av_ versus log M_r_) derived from the standard proteins (Figure 5C), the native size of GabR was calculated to be 420 kDa. Since the theoretical mass of GabR is 55.75 kDa, the native size suggests that *C. glutamicum* GabR forms an octamer (theoretical mass 446 kDa).

### Determination of Transcriptional Start Sites

**FIGURE 6:**
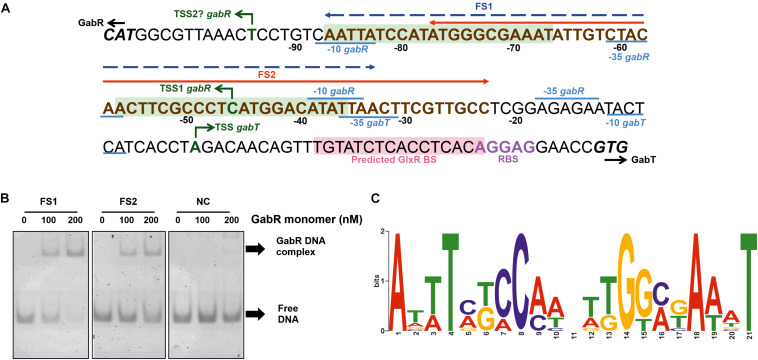
Determination of the binding site(s) of GabR in the *gabTDP* promoter region. **(A)** Sequence of the *gabR-gabT* intergenic region. The ATG start codon of *gabR* (cg0565) and the GTG start codon of *gabT* (cg0566) are shown in bold and italic. The transcriptional start sites of *gabR* and *gabT* are indicated with green arrows. The corresponding –10 and –35 regions are labeled in blue. The ribosome binding site of *gabTDP* is labeled in purple, and the predicted GlxR binding site is marked with light red background. The binding region for GabR determined in this work by EMSAs is marked in bold brown letters (compare Supplementary Figure 3). Fragments FS1 (–87 to –33) and FS2 (–77 to –23) are marked in the figure by a blue or red arrow, respectively. Potential binding sites for GabR are marked with light green background. **(B)** EMSAs with purified GabR and DNA fragments FS1 and FS2. The fragments were incubated with the indicated GabR concentrations (given in nM of monomers). A 55 bp fragment downstream of the predicted binding sites was used as negative control (NC). **(C)** Proposed GabR consensus binding site identified with MEME software using the *gabR*-*gabT* intergenic regions of *C. glutamicum* WT, *Corynebacterium deserti* and *Corynebacterium callunae* as input sequences.

In a previous RNAseq study, a TSS of P*_*gabR*_* was identified, but no TSS of P*_*gabTDP*_*, presumably because the latter genes were not expressed in the absence of GABA (Pfeifer-Sancar et al., 2013). Therefore, we determined the TSS of P*_*gabTDP*_* initially using a 5′/3′-RACE kit and identified a single TSS for *gabT* located 36 bp upstream of the *gabT* start codon (Figure 6A). The -10 region (TACTCA) and the -35 region (TTAACT) of P*_*gabTDP*_* and the RBS of *gabT* (AGGAG) were predicted according to known consensus sequences (Pfeifer-Sancar et al., 2013). In an alternative approach, we performed RNAseq of GABA-grown WT cells to identify TSSs. For *gabT*, the transcriptional start site found by 5′/3′-RACE was confirmed with 43906 reads starting at this position. Interestingly, another TSS was identified within the *gabD* coding region. 28 reads started 210 bp upstream of the *gabP* start codon, suggesting the presence of an additional weak promoter for *gabP* expression. The deduced -10 region (AACAAT) and -35 region (TTCGGC) showed reasonable similarity to the reported consensus sequences (Pfeifer-Sancar et al., 2013). This promoter might allow GabR-independent weak expression of *gabP* and could enable initial GABA uptake to allow GabR-dependent expression of the *gabTDP* operon. With respect to the TSS of *gabR*, the RNAseq data revealed only two reads that started at the position 59 bp upstream of the *gabR* start codon (Figure 6A) that was previously reported (Pfeifer-Sancar et al., 2013). Four reads started 11 bp upstream of the *gabR* start codon, suggesting either another very weak promoter for *gabR* expression with weakly conserved -10 region (TAATTG) and -35 region (TTGTAG) or processing of the transcript starting 59 bp upstream of the *gabR* start codon.

### Determination of the GabR Binding Site(s) in the *gabTDP* Promoter Region

For the determination of the GabR binding site, EMSAs were performed with purified GabR. In the first experiment, binding of GabR to a 500 bp fragment covering the entire *gabR*-*gabT* intergenic region was analyzed using a DNA fragment of similar size of the *ldhA* gene of *C. glutamicum* as negative control. The *gabR*-*gabT* intergenic region was shifted partially with 80 nM GabR (monomer) and fully with 240 nM GabR (Supplementary Figure 2), whereas no shift was observed for the control fragment up to 320 nM GabR (Supplementary Figure 2). This suggests that GabR binds specifically to the *gabTDP* promoter region. In the following, we reduced the size of the DNA fragments step by step to localize the binding site(s) of GabR using the above mentioned fragments as positive and negative controls. As shown in Supplementary Figure 3, a full shift was observed for fragments FA1 and FA2, a partial shift for FA3, and a very poor shift for FA4 and FA5. For the fragments starting at the other side of the promoter region, clear shifts were observed for fragments FB1, FB2 and FB3, weak shifts for FB4 and FB5, and no shift for FB6. These results suggested that GabR binds between position −87 and −23 with respect to the TSS of *gabT*.

Regulator binding sites are often conserved among closely related species, which is useful to identify a binding motif when only a single target promoter is known (Wennerhold et al., 2005). We searched for a binding motif in the *gabR*-*gabT* intergenic regions of *C. glutamicum, Corynebacterium deserti*, and *Corynebacterium callunae* using MEME software (Bailey et al., 2006). The search uncovered a 21 bp inverted repeat that was present 1-2 times in each input sequence (Figure 6C). An alignment of the input sequences (Supplementary Figure 4) revealed that the regions encompassing the two proposed GabR binding motifs belong to the more conserved regions, which is a further hint that these represent GabR binding sites.

In further EMSAs, we used 55 bp fragments to verify the localization of the GabR binding sites in the *C. glutamicum gabTDP* promoter. FS1 is a fragment covering both of the predicted binding sites, whereas FS2 covers half of the first binding site and the entire second binding site (Figure 6A). A 55 bp fragment downstream of the predicted binding sites was used as negative control. Binding of GabR was observed for both FS1 and FS2, but it was stronger for FS1 (Figure 6B). This suggests that one complete binding site is sufficient for weak binding, but two sites are required for strong binding by GabR. In another experiment, binding of GabR to two 31 bp fragments (FS3 and FS4) covering one binding site each was tested. A 31 bp fragment downstream of the predicted binding sites was used as negative control. Reasonable binding was observed for FS3 containing the first binding site and weak binding, not much stronger compared to the negative control, was observed for FS4 (Supplementary Figure 5). The band of the complex did not run far into the gel, presumably because of the large size of the GabR octamer. We assume that this band represents the complex, as it was absent from the negative control and from the DNA samples without protein. Our results suggest that each of the two binding sites can be bound separately by GabR, but binding is much stronger when both binding sites are present.

To verify that both binding sites are relevant for good binding, we mutated four conserved bases of both motifs either separately or in combination in fragment FS1 and analyzed the binding by GabR (Supplementary Figure 6). Mutation of one of the binding sites reduced the affinity to GabR, and with both binding sites mutated, the binding was almost completely abolished. This verifies that both sites are relevant for GabR binding.

### Influence of Ammonium and Urea on Growth of *C. glutamicum* With GABA

**FIGURE 7:**
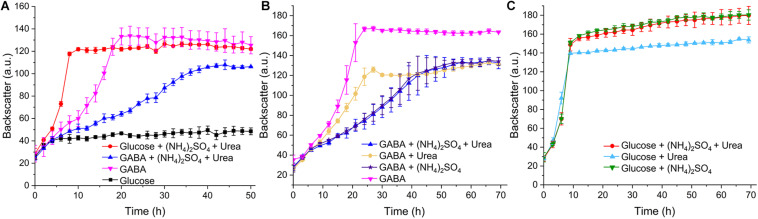
Growth of *C. glutamicum* WT with different carbon and nitrogen sources. Cells were precultured in BHIS medium for 12 h and subsequently washed with phosphate buffer before inoculation of the main cultures to a starting OD_600_ of 0.5. The main cultivation was performed in a BioLector microcultivation system with 48-well Flower plates each containing 750 μL CGXII minimal medium supplemented with 41.7 mM glucose or 62.5 mM GABA. 151 mM (NH_4_)_2_SO_4_ or 83 mM urea were included as indicated in panels **(A–C)**. Mean values and standard deviations of three biological replicates are shown.

In our initial growth studies using the BioLector microcultivation system, *C. glutamicum* WT was cultivated in standard CGXII minimal medium containing either glucose (41.7 mM corresponding to 250 mM carbon) or GABA (62.5 mM corresponding to 250 mM carbon) as carbon sources. In glucose-containing medium, a growth rate μ of 0.4 h^–1^ was observed, whereas it was only 0.05 h^–1^ in GABA-containing medium. Interestingly, a growth rate of 0.13 h^–1^ was obtained when the standard nitrogen sources of CGXII medium, ammonium sulfate (151 mM) and urea (83 mM), were omitted and GABA served as sole carbon and nitrogen source (Figure 7A). Measurement of the final OD_600_ in a spectrophotometer after 50 h revealed no significant difference between the cultures with glucose, (NH_4_)_2_SO_4_ and urea on the one hand (OD_600_ = 12.17 ± 0.54) and the cultures with GABA without additional nitrogen sources on the other hand (OD_600_ = 11.81 ± 0.96). Further growth experiments revealed that (NH_4_)_2_SO_4_ alone was sufficient to exert the same inhibitory effect on growth with GABA (μ = 0.04 h^–1^) as in combination with urea (μ = 0.05 h^–1^) (Figure 7B). Urea alone was also inhibitory, but to a lesser extent (μ = 0.08 h^–1^). Urea is converted by urease to ammonium and carbon dioxide (Siewe et al., 1998), but the ammonia levels formed in this way are probably lower than in media containing (NH_4_)_2_SO_4_, which could explain the weaker inhibitory effect of urea. When similar experiments were performed with glucose as carbon source, all cultures grew with the same rate (Figure 7C). A further experiment revealed that the inhibitory effect of (NH_4_)_2_SO_4_ on growth with GABA was concentration-dependent and weak inhibition was observed already with 10 mM (NH_4_)_2_SO_4_ (Supplementary Figure 7). The negative influence of 151 mM (NH_4_)_2_SO_4_ on the final backscatter during growth with GABA as sole carbon source varied throughout our study. Sometimes the same final backscatter was reached in the presence of (NH_4_)_2_SO_4_ as in its absence, whereas in other experiments the final backscatter was much lower in the presence of (NH_4_)_2_SO_4_. The reason for this variability is currently unknown. In summary, *C. glutamicum* grew quite well in minimal medium with GABA as sole carbon and nitrogen source and the additional presence of ammonium was inhibitory. In the following studies, we searched for the basis of ammonium inhibition.

### Influence of Ammonium on GABA Consumption

**FIGURE 8:**
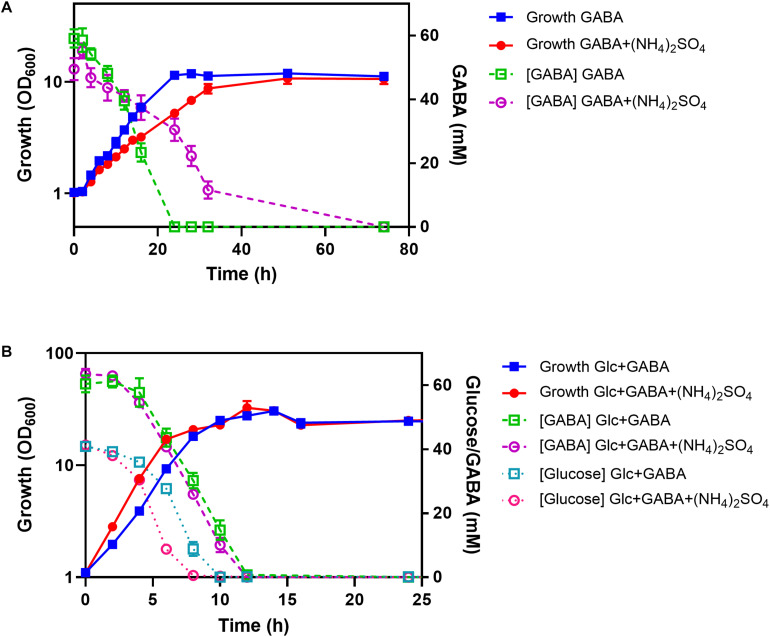
Influence of (NH_4_)_2_SO_4_ on GABA consumption in the absence **(A)** and presence **(B)** of glucose (Glc). *C. glutamicum* WT was cultured in shake flasks containing CGXII medium with the indicated carbon and nitrogen sources (62.5 mM GABA, 41.7 mM glucose, 151 mM (NH_4_)_2_SO_4_). Glucose and GABA concentrations were determined by HPLC. Mean values and standard deviations of three biological replicates are shown.

To test the influence of ammonium on GABA consumption, the WT was cultured in shake flasks with minimal medium containing either GABA or GABA plus (NH_4_)_2_SO_4_. With GABA as sole carbon and nitrogen source, a growth rate of 0.12 h^–1^ was observed, which was reduced to 0.08 h^–1^ in the presence of 151 mM (NH_4_)_2_SO_4_ (Figure 8A). In the absence of (NH_4_)_2_SO_4_, GABA (62.5 mM) was completely consumed after 24 h and only after about 36 h in the presence of (NH_4_)_2_SO_4_. Both cultures reached a final OD_600_ of about 11, corresponding to 2.75 g cell dry weight/L (Kabus et al., 2007). In a further experiment, cells were cultivated in minimal medium containing either GABA and glucose or GABA, glucose, and (NH_4_)_2_SO_4_. In this case, the cultures with (NH_4_)_2_SO_4_ grew slightly faster (μ = 0.46 h^–1^) than the ones without (NH_4_)_2_SO_4_ (μ = 0.39 h^–1^) and also consumed glucose faster than the cultures without (NH_4_)_2_SO_4_ (Figure 8B). Complete glucose consumption was observed after 8 h and 10 h, respectively. Interestingly, GABA consumption was comparable for the cultures with and without (NH_4_)_2_SO_4_, in contrast to the cultures without glucose, and complete GABA consumption was observed after 12 h. It is obvious from Figure 8B that glucose and GABA are used simultaneously, also in the presence of (NH_4_)_2_SO_4_.

### Relevance of Ammonia-Assimilating Enzymes for Growth With GABA

During growth in minimal medium with GABA as sole carbon and nitrogen source, L-glutamate is formed from 2-oxoglutarate by the GABA transaminase GabT. L-Glutamine synthesis is dependent on glutamine synthetase encoded by the *glnA* gene (Jakoby et al., 1997). A second putative glutamine synthetase is encoded by *glnA2*, but this gene cannot complement the glutamine auxotrophy of a Δ*glnA* mutant and the function of the GlnA2 protein is not known yet (Nolden et al., 2001; Rehm and Burkovski, 2011). Glutamine synthetase requires L-glutamate, ATP and NH_4_^+^ as substrates. Therefore, the question arises which reactions lead to ammonia release and thereby allow glutamine synthesis during growth with GABA as sole nitrogen source. A reasonable candidate is glutamate dehydrogenase (Gdh), which catalyzes the reductive amination of 2-oxoglutarate with ammonia and NADPH to L-glutamate and NADP^+^, and also the reverse reaction (Shiio and Ozaki, 1970). In order to determine whether Gdh is important as provider of ammonium during growth on GABA, we tested a Δ*gdh* mutant and found that it grew similarly as the wild type with GABA as sole carbon and nitrogen source (data not shown). This does not exclude the Gdh reaction as a source of ammonia, but shows that alternative ways for ammonium generation exist.

**FIGURE 9:**
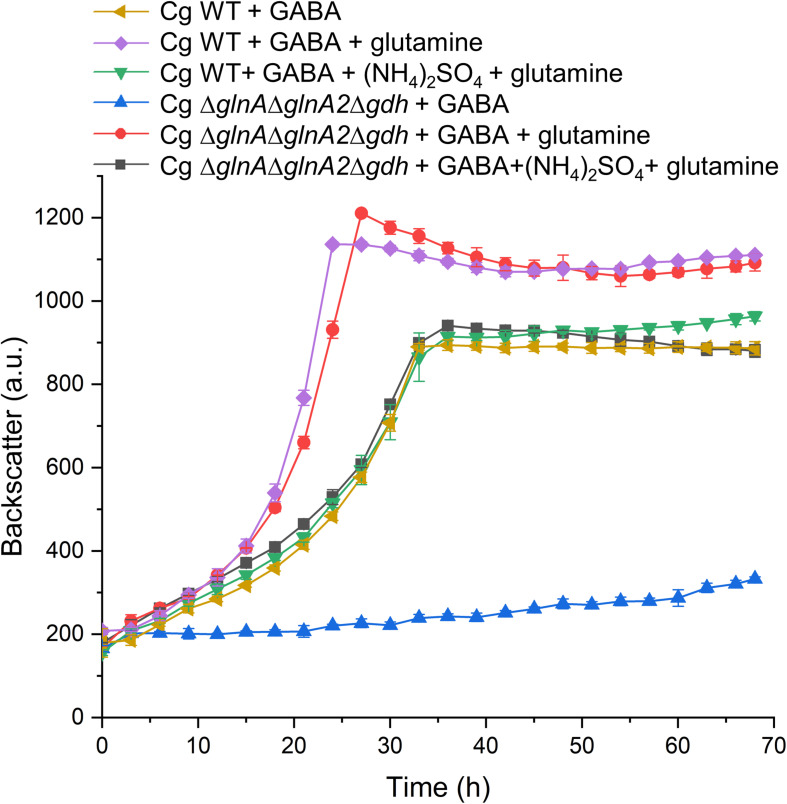
Influence of L-glutamine supplementation on growth of *C. glutamicum* WT and the Δ*glnA*Δ*glnA2*Δ*gdh* mutant in CGXII medium with GABA. Cells were precultured in BHIS medium for 12 h and subsequently washed with phosphate buffer before inoculation of the main cultures to a starting OD_600_ of 0.5. The main cultivation was performed in a BioLector microcultivation system with 48-well Flower plates each containing 750 μL CGXII minimal medium supplemented with 62.5 mM GABA. 20 mM L-glutamine and 151 mM (NH_4_)_2_SO_4_ were included as indicated. For the WT mean values and standard deviations of three biological replicates are shown, for the mutant mean values and standard deviations of two biological replicates.

Besides the Δ*gdh* mutant, we also tested the growth behavior on GABA of other mutants with defects in nitrogen assimilation, which are mutants lacking either glutamate synthase (Δ*gltBD*), or glutamine synthetase (Δ*glnA*), or a triple mutant devoid of GlnA, GlnA2, and Gdh (Δ*glnA*D*glnA2*D*gdh*). Whereas the Δ*gltBD* mutant grew like the WT, the other two mutants were unable to grow with GABA as sole carbon and nitrogen source, irrespective of the absence or presence of ammonium. In Figure 9, the data for the triple mutant are shown. The result corresponds to the expectation that GltBD is not required for glutamate synthesis, whereas GlnA is essential for glutamine synthesis. This was further confirmed by supplementing GABA medium with L-glutamine, which rescued growth of the triple mutant (Figure 9). The addition of L-glutamine also improved the growth rate of the WT with GABA by about 50% (μ = 0.15 h^–1^ vs. μ = 0.10 h^–1^ without glutamine) and the final backscatter by about 25% (Figure 9). *C. glutamicum* is able to use L-glutamine as sole carbon and nitrogen source (Rehm et al., 2010). Whereas the increased final backscatter can be explained by the use of L-glutamine as additional carbon source, the increased growth rate suggests a deficiency in glutamine availability when GABA serves as sole nitrogen source. Interestingly, when (NH_4_)_2_SO_4_ was added to medium with GABA and glutamine, it again had a negative effect on growth of both the WT and the triple mutant (Figure 9). This suggests that the inhibitory effect of ammonium on growth with GABA alone is not due to a negative effect on glutamine synthesis.

### Influence of Growth Conditions and GabR on the Promoter Activity of P*_*gabTDP*_*

**FIGURE 10:**
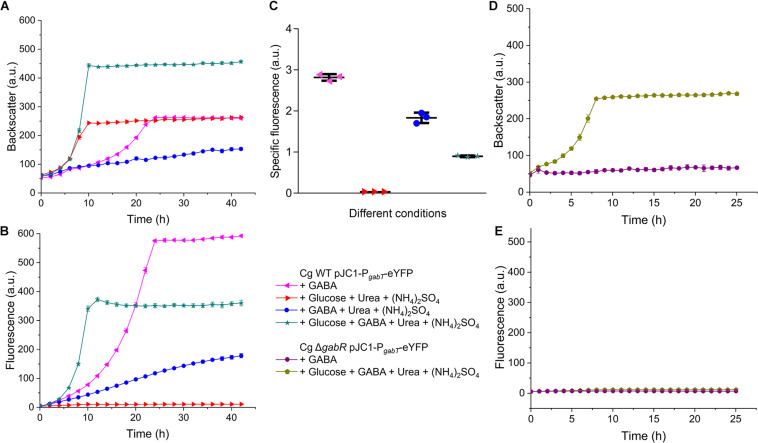
Relevance of GabR for *gabTDP* transcription. *C. glutamicum* WT or the Δ*gabR* mutant transformed with the reporter plasmid pJC1-P*_*gabT*_*-eYFP were precultivated in BHIS medium and washed with phosphate buffer before inoculation of the main cultures. The cultivations were performed in a BioLector microcultivation system at 30°C and 1200 rpm using 750 μl CGXII minimal medium supplemented with GABA (62.5 mM), glucose (41.7 mM), (NH_4_)_2_SO_4_ (151 mM), and urea (83 mM) as indicated. **(A,B)** Growth and eYFP fluorescence representing the activity of the *gabTDP* promoter of *C. glutamicum* WT with pJC1-P*_*gabT*_*-eYFP. **(C)** Specific fluorescence (ratio fluorescence/backscatter) of the cultures shown in **(A)** and **(B)** after 40 h. **(D,E)** Growth and fluorescence *C. glutamicum* Δ*gabR* harboring pJC1-P*_*gabT*_*-eYFP. Mean values and standard deviations of three biological replicates are shown.

To analyze the influence of ammonia and other conditions on the expression of the *gabTDP* operon, we constructed the reporter plasmid pJC1-P*_*gabT*_*-eYFP containing a transcriptional fusion between P*_*gabTDP*_* (500 bp upstream of the *gabT* start codon) and the eYFP-encoding gene. Thereby the promoter activity can be monitored by measuring the fluorescence of the culture. Cell density (backscatter) and eYFP fluorescence of WT cells with pJC1-P*_*gabT*_*-eYFP cultivated in different media is shown in Figures 10A,B. As the cells reached different final backscatter values, the specific fluorescence (representing the ratio of fluorescence/backscatter) was calculated for the cultures in the stationary phase at 40 h, which represents the activity of P*_*gabTDP*_* over the entire cultivation (Figure 10C). We also checked the specific fluorescence of the cultures in the exponential growth phase, which was very similar to the values after 40 h (data not shown). Maximal specific fluorescence (2.81 ± 0.07) was observed for the cultures grown with GABA alone, whereas it was almost zero (0.03 ± 0.00) for the cultures grown with glucose alone, indicating that P*_*gabTDP*_* was active only in the presence of GABA. The specific fluorescence of the cultures grown with GABA, ammonium sulfate and urea (1.84 ± 0.10) was reduced by 35% in comparison to the cultures with GABA alone, indicating that ammonium inhibited transcription of *gabTDP.* Addition of glutamine to a culture with GABA and ammonium sulfate did not enhance *gabTDP* expression (data not shown), suggesting that glutamine does not influence transcription of *gabTDP*.

The specific fluorescence of cells cultivated with GABA, glucose, ammonium sulfate and urea (0.90 ± 0.02) was reduced by even 68% compared to growth on GABA alone, indicating that not only ammonium, but also glucose had a negative effect on *gabTDP* transcription. This effect was not specific for glucose, as it was also observed with gluconate or *myo*-inositol as additional carbon sources (Supplementary Figure 8).

In further experiments, we analyzed the activity of P*_*gabTDP*_* in the Δ*gabR* mutant (Figures 10D,E). In medium with only GABA as carbon and nitrogen source, the mutant showed neither growth nor fluorescence, as expected. In medium with glucose and GABA, the mutant was able to grow, but no fluorescence was observed. This result confirms that besides GABA also GabR is essential for expression of the *gabTDP* genes.

### Influence of Plasmid-Based *gabTDP* Expression on the Effect of Ammonium

**FIGURE 11:**
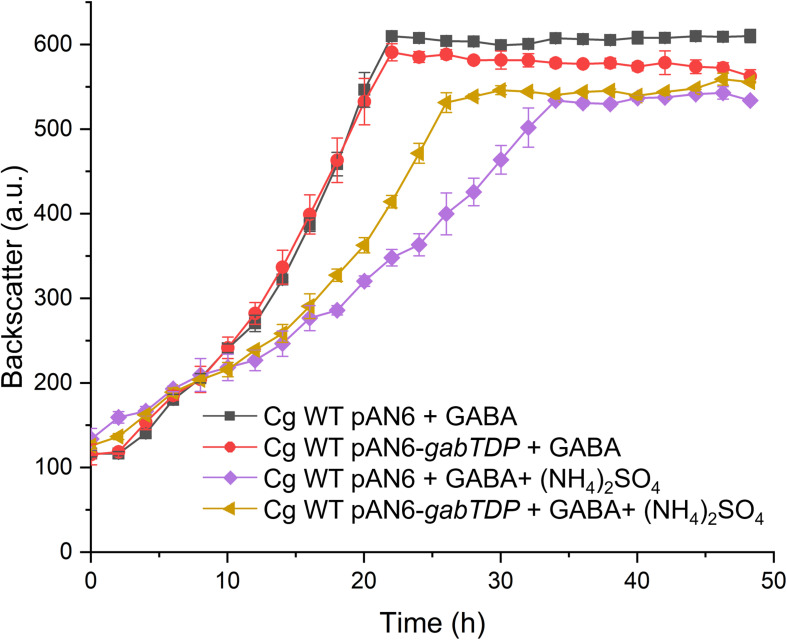
Growth of *C. glutamicum* WT harboring pAN6-*gabTDP* or pAN6 with different substrates in the presence of 10 μM IPTG. Growth experiments were performed in a BioLector microcultivation system with 48-well Flower plates containing 750 μL CGXII minimal medium supplemented with GABA (62.5 mM) and (NH_4_)_2_SO_4_ (151 mM) as indicated. BHIS medium was used for precultures and the cells were washed with phosphate buffer before inoculation of the main culture. Mean values and standard deviations of three biological replicates are shown.

The reporter gene assays had revealed that ammonium reduces transcription of *gabTDP* (Figure 10C) by about 35%. To test whether this effect is sufficient to explain the growth inhibition by ammonium, we analyzed the consequences of plasmid-based overexpression of *gabTDP*. *C. glutamicum* WT was transformed with pAN6-*gabTDP* or pAN6 as control. The two strains were cultivated with GABA alone or with GABA plus (NH_4_)_2_SO_4_ and expression of plasmid-borne *gabTDP* was induced with 10 μM IPTG (Figure 11). With GABA alone, the WT containing pAN6-*gabTDP* grew comparably to the control strain with pAN6, suggesting that under these conditions chromosomal *gabTDP* expression was not limiting growth. In medium with GABA and (NH_4_)_2_SO_4_, the strain with pAN6-*gabTDP* grew faster than the control strain with pAN6, although still not as good as with GABA alone, even when *gabTDP* expression was induced with 100 μM IPTG (data not shown). The results indicate that increased expression of *gabTDP* can partially overcome the growth inhibition by (NH_4_)_2_SO_4_.

## Discussion

In this study, we analyzed the regulation of GABA utilization in the actinobacterial model organism *C. glutamicum*. The fact that *C. glutamicum* possesses the genes/proteins required for GABA uptake and metabolism suggests that GABA occurs in its natural soil habitat, where it could be derived e.g., from decomposed plant material or formed by other microorganisms via glutamate decarboxylation. We show that expression of the *gabTDP* operon encoding the proteins involved in GABA uptake and catabolism is transcriptionally activated by the divergently encoded regulator GabR. GabR is essential for growth of *C. glutamicum* on GABA. The observation that growth of a Δ*gabR* mutant could be restored by plasmid-based, GabR-independent expression of the *gabTDP* genes indicated that no additional genes activated by GabR are required for GABA utilization, characterizing GabR as a local regulator.

GabR belongs to the PucR family of transcriptional regulators (Pfam PF07905). According to Pfam, more than 95% of the PucR sequences in the database are derived from *Actinobacteria* or *Firmicutes*. About half of the PucR family members in the database contain a GGDEF-like domain. Only few PucR-type regulators were studied to date, all of which contain the GGDEF-like domain, but a specific function of this domain has not been reported. These regulators are *B. subtilis* PucR involved in purine catabolism (Beier et al., 2002), *B. subtilis* PutR involved in proline utilization (Belitsky, 2011), *B. subtilis* AdeR involved in alanine catabolism (Lin et al., 2012), *Streptomyces ambofaciens* Srm22 (SrmR) controlling expression of a polyketide synthase gene for the synthesis of an antibiotic (Geistlich et al., 1992; Karray et al., 2010), and *Escherichia coli* CdaR (SdaR), which regulates genes involved in the uptake and metabolism of galactarate and glucarate (Monterrubio et al., 2000). All of these PucR regulators are activators, except for *B. subtilis* PucR, which acts both as activator and as repressor. GabR from *C. glutamicum* is the first characterized member of the PucR family that does not contain a GGDEF-like domain. Our results suggest that GabR binds to two 21 bp inverted repeats in the *gabTDP* promoter region. For AdeR, a 24 bp inverted repeat and for PutR, a 17 bp inverted repeat have been determined as binding sites, which is similar to our findings (Belitsky, 2011; Lin et al., 2012). The binding site of PucR is not palindromic (Beier et al., 2002) and the binding sites for Srm22 and CdaR have not been determined yet.

Gel filtration of GabR indicated that it forms octamers. As the oligomeric state of other PucR-like regulators has not been determined yet, we do not know whether this is a specific feature of this group of regulators. We searched for other octameric transcriptional regulators to compare their binding patterns and function. The LysR-type transcriptional regulator (LTTR) CrgA of *Neisseria meningitides* forms octameric rings and is proposed to bind to two binding sites in its target promoter with two stacked octameric rings (Sainsbury et al., 2009). The two CrgA binding sites cover a region of 63 bp, which is quite similar in size to the region covered by our two proposed motifs. CrgA also binds to DNA fragments containing just one of the binding sites (Sainsbury et al., 2009), but with strongly decreased affinity, which is also comparable to our data. GabR might work in a similar manner. Regulators belonging to the Lrp family are also frequently forming octamers, for example Lrp of *E. coli* (de los Rios and Perona, 2007), LrpA of *Pyrococcus furiosus* (Leonard et al., 2001), LrpA of *Mycobacterium tuberculosis* (Reddy et al., 2008; Song et al., 2016), AldR of *M. tuberculosis* (Dey et al., 2016), or BarR of the archeum *Sulfolobus acidocaldarius* (Liu et al., 2014).

Transcriptional activators for catabolic pathways often require the corresponding substrate or one of its degradation products as co-activator. In the case of GabR from *C. glutamicum*, binding to its target DNA did not require the presence of GABA and the presence of GABA had no obvious influence on the binding affinity (Supplementary Figure 9). GabR might bind to its target DNA both in the apo-state and in the ligand-bound state, but requires binding of GABA or another effector metabolite to trigger a conformational change that is necessary to activate transcription of *gabTDP*. Such a situation was found e.g., for *B. subtilis* GabR_Bs_, which binds to its target promoter independent of GABA, but transcription activation of the target genes requires binding of GABA and PLP (Wu et al., 2017). However, GabR_Bs_ belongs to a different protein family than GabR of *C. glutamicum* and also PLP had no influence on GabR binding to DNA, neither alone nor in combination with GABA (Supplementary Figure 9). In case of *B. subtilis* PutR, the presence of proline increased the affinity for its target DNA about 10-fold (Belitsky, 2011), whereas for *B. subtilis* AdeR, L-alanine did not increase the affinity for the target promoter (Lin et al., 2012). Further studies are required to identify the co-activator(s) of GabR and the mechanism of transcriptional activation.

The GabR binding site is located immediately upstream of the -35 region of P*_*gabTDP*_* (Figure 6), which is a typical position for transcriptional activators. However, in the transcriptome analysis, not only the *gabTDP* mRNA levels were increased during growth with GABA, but also the *gabR* mRNA level, although to a much lesser extent (4-fold) compared to *gabTDP* (65- to 87-fold). As the GabR binding site is located downstream of the -10 region of the *gabR* promoter identified in previous RNAseq studies (Pfeifer-Sancar et al., 2013), the question arises how the apparent positive autoregulation of *gabR* is accomplished. One possibility could be an alternative transcriptional start site, leading e.g., to a leaderless transcript, which is found quite often in *C. glutamicum*. Our RNAseq analysis indeed identified a possible alternative TSS located 11 bp upstream of the *gabR* start codon (Figure 6A), but also in this case the promoter region overlaps with one of the two GabR binding sites.

When GABA is used as sole carbon source by *C. glutamicum*, growth requires the formation of PEP for gluconeogenesis. This task is fulfilled by PEP carboxykinase encoded by the *pck* gene (Riedel et al., 2001). Expression of the *pck* gene was found to be slighly upregulated in cells grown with GABA (1.68-fold, *p*-value 0.02), supporting its requirement in gluconeogenesis. Transcription of *pck* is subject to regulation by at least four transcriptional regulators and upregulation during growth on GABA might be due to derepression by RamA or activation by GntR1/GntR2 (Klaffl et al., 2013).

We observed that ammonium inhibits growth with GABA, with 10 mM already showing slight growth retardation. This negative effect is caused at least in part by an influence on *gabTDP* transcription, which was reduced by about 35% in the presence of 151 mM ammonium and 83 mM urea (Figure 10). The molecular basis for the diminished expression is not known yet. Control of nitrogen metabolism has been extensively studied in *C. glutamicum* and AmtR was identified as global regulator (Jakoby et al., 2000; Beckers et al., 2005; Buchinger et al., 2009). In the presence of sufficient nitrogen, AmtR represses a large set of genes involved in uptake and utilization of various nitrogen sources. However, the *gabTDP* operon or *gabR* were never identified as members of the AmtR regulon, arguing against AmtR as mediator of reduced *gabTDP* expression in the presence of ammonia. In our EMSA studies with purified GabR, ammonia did not influence binding to the *gabT* promoter region (Supplementary Figure 10), but this does not exclude an effect of ammonia on GabR-activated transcription initiation. For example, binding of ammonium to GabR might inhibit binding of GABA or another effector and thus transcriptional activation. Future studies are required to clarify the mechanism by which ammonium inhibits *gabTDP* expression.

Growth with GABA as sole carbon and nitrogen sources requires the formation of ammonium as substrate for glutamine synthetase. Oxidative deamination of glutamate by glutamate dehydrogenase might contribute to ammonium provision, although deletion of the *gdh* gene had no effect on growth with GABA. An alternative possibility is the reaction catalyzed by aspartate ammonia-lyase, which catalyzes the conversion of aspartate to fumarate and ammonia (Dover and Halpern, 1972). The corresponding gene *aspA* (cg1697) was 2.50-fold upregulated in cells grown with GABA and urea compared to cells grown with glucose and urea. The observation that glutamine supplementation improved the growth rate with GABA suggests that glutamine availability in the absence of supplementation is limited, presumably by limited availability of the ammonia for glutamine synthetase. However, glutamine stimulated growth also in the presence of ammonium. In this situation, glutamine synthetase activity might be limiting due to limited expression of the *glnA* gene in the presence of ammonium (Nolden et al., 2001; Strösser et al., 2004) and due to limited activity of the enzyme due to adenylylation by GlnE (Amon et al., 2010; Rehm and Burkovski, 2011). We also tested whether ammonium inhibits the activity of the GabT transaminase activity using purified GabT, but observed only a slight reduction by 6% in the presence of 30 mM (NH_4_)_2_SO_4_ (9.07 ± 0.33 μmol min^–1^ mg^–1^ vs. 9.69 ± 0.12 μmol min^–1^ mg^–1^ in the absence of ammonium sulfate).

Besides ammonium, also the presence of alternative carbon sources had a negative influence on the expression of the *gabTDP* operon. The inhibition by glucose, gluconate, and *myo*-inositol is presumably caused by repression of *gabTDP* by the global cAMP-dependent transcriptional regulator GlxR. GlxR can function both as activator and repressor, depending on the localization of the binding site. A GlxR binding site (5′-TGTATCTCACCTCACA-3′) has been detected in the *gabR-gabT* intergenic region (Toyoda et al., 2011; Jungwirth et al., 2013), which is located downstream of the *gabT* TSS (Figure 6A), indicating a repressor function of GlxR for *gabTDP*. GlxR-binding to DNA is controlled by cAMP, which is formed by the membrane-bound adenylate cyclase CyaB (Cha et al., 2010; Wolf et al., 2020). The tested additional carbon sources might lead to elevated cAMP levels and thus to enhanced binding of GlxR to the *gabT* promoter and repression. In contrast to e.g., *E. coli*, *C. glutamicum* usually shows parallel consumption of carbon sources rather than diauxic growth. This has been demonstrated e.g., for glucose and acetate (Wendisch et al., 2000), glucose and gluconate (Frunzke et al., 2008), or glucose and citrate (Brocker et al., 2009). GlxR is presumably involved in coordinating the consumption of carbon sources in *C. glutamicum* to avoid an overload of the metabolic capacity of the cells. In the case of GABA, a similar situation can be envisaged, where the presence of an alternative carbon source causes a reduction of the GABA utilization rate to avoid an overload of the TCA cycle at the stage of succinate.

In summary, we characterized GabR as the first PucR-type transcriptional regulator without a GGDEF-like domain and showed that GabR is essential for activating expression of the GABA catabolic genes *gabTDP*. Although GABA did not influence the binding affinity of purified GabR to its target promoter, we assume it to be the most likely effector molecule, as the *gabTDP* genes were not expressed in the absence of GABA. The inhibitory effect of ammonium on GABA utilization is caused via inhibition of *gabTDP* transcription via a yet unknown mechanism and presumably via a negative effect on glutamine synthetase expression and activity. The negative effect on *gabTDP* transcription by alternative carbon sources is likely caused by the global cAMP-dependent regulator GlxR. Many of the results obtained here will probably also be relevant for other *Actinobacteria* capable of utilizing GABA as carbon and nitrogen source.

## Orcid

Lingfeng Zhu

orcid.org/0000-0002-9145-5419

Christina Mack

orcid.org/0000-0002-3495-3796

Astrid Wirtz

orcid.org/0000-0003-2159-3717

Angela Kranz

orcid.org/0000-0002-8000-0400

Tino Polen

orcid.org/0000-0002-0065-3007

Meike Baumgart

orcid.org/0000-0002-9874-1151

Michael Bott

orcid.org/0000-0002-4701-8254

## Data Availability Statement

The microarray datasets generated for this study can be found in the NCBI Gene Expression Omnibus under the GEO accession number GSE138829. The RNAseq data are available in the GEO database with the accession number GSE156688.

## Author Contributions

LZ performed the majority of the experimental work presented in this study, wrote the first draft of the manuscript and provided most of the figures and tables. CM performed several growth experiments in shake flasks and the BioLector microcultivation system and she was responsible for the RNA isolation for RNAseq analysis. AW established and performed the HPLC method for measuring the GABA concentration in supernatants. AK evaluated the RNAseq data and TP supported the DNA microarray analysis. MBa and MBo designed the study, supervised the experimental work, and evaluated the data together with LZ, AK, and TP. MBa prepared figures added during revision. MBa and MBo revised the manuscript and MBo was responsible for the final version. All authors contributed to the article and approved the submitted version.

## Conflict of Interest

The authors declare that the research was conducted in the absence of any commercial or financial relationships that could be construed as a potential conflict of interest.

## Footnotes

1 http://www.bioconductor.org
