# Supplementary material for: Regulation of γ-Aminobutyrate (GABA) Utilization in Corynebacterium glutamicum by the PucR-Type Transcriptional Regulator GabR and by Alternative Nitrogen and Carbon Sources
Source: Front Microbiol. 2020 Oct 27;11:544045. doi: 10.3389/fmicb.2020.544045 (PMC7652997; doi:10.3389/fmicb.2020.544045)
Supplement: Supplementary file 1 [file Data_Sheet_1.PDF]

frontiers

Supplementary Material

1 Supplementary Tables

TABLE S1. Oligonucleotides used in this study.

Oligonucleotide	Sequence (5' → 3') and properties[a]
**Construction of deletion plasmid pK19mobsacB-Δ*gabR* and PCR analysis of the resulting mutants**	
cg0565frontF	**ATGCCTGCAGGTCGACTCTAGAGGATC**TGAGGCTGCTGGCGCTGACTTCGTT
cg0565frontR	**CGCCCATATGGATAATTGACAGGAGTTTAACGCCAAAGACCCCATGCAAAGCATGGGGCC**
cg0565backF	**GGCCCCATGCTTTGCATGGGGTCTTTGGCGTTAAACTCCTGTCAATTATCCATATGGGCG**
cg0565backR	**CACGACGTTGTAAAACGACGGCCA**GTGAATTGTGATCGCCTGGAGTCAAGGCGTTGAG
cg0565checkF	CAAGAACTACGACGCTTCCATCGAC
cg0565checkR	AGTGGTCCGAATCCGGACTTGTATG
**Construction of plasmid pAN6-*gabR***	
0565F	GAACATATGGAAACCCCAACCCAAGACAT
0565R	GAAGCTAGCTTATTGCTTTTCGACGTCTC
**Construction of plasmid pET-TEV-*gabR***	
0565F	GAACATATGGAAACCCCAACCCAAGACAT
petfhis0565R	CATCCAAGCTTATTGCTTTTCGACGTCTC
**Construction of plasmid pJC1-P_{gabT}-eYFP**	
PgabTF	**GATCAGCGACGCCGCAGGGGGATC**ATTTCACGGGGGACTTCAAAGA
PgabtR	**CAGCTCCTCGCCCTTGCTCACCAT**GGTTCCTCCTGTGAGGTGAGATAC
eYFPF	**ATGGTGAGCAAGGGCGAGGAGCTG**TTCA
eYFP-termR	**GTAAAACGACGGCCAGTACTAGT**TTATCTAGACTTGTACAGCTCGTCCATGCCG
**Construction and sequencing of plasmid pAN6-*gabTDP***	
gabtdpF	**CATGCCTGCAGAAGGAGATATACAT**ATGGAAGATCTCTCATACCGCATC
gabtdpR	**GAACTGTGGGTGGGACCAGCTAGC**CTATGCCCAACCCGCAGGCATTAATC
gabtdpS2	CCAGGTCGGAGCCGAAAACCTCGC
gabtdpS3	CAGGTGTTAGTCGCAGCCCTAGAG
gabtdpS4	CTGATCCTCGCTTGGCTAAAGTCA
gabtdpS5	CAATCCGGCTTCGGCAGAGAAGGC
**Sequencing primers for plasmid pK19mobsacB**	
pK19F	CGCCAGGGTTTTCCCAGTCAC

Oligonucleotide	Sequence (5' → 3') and properties[a]
pK19R	AGCGGATAACAATTTCACACAGGA
**Sequencing primers for plasmid pAN6**	
pAN6F	GATATGACCATGATTACGCCAAGC
pAN6R	GACCGCTTCTGCGTTCTGATTTAA
**Sequencing primers for plasmid pET-TEV**	
pET-TEVF	GAAACAAGCGCTCATGAGCCCGAAG
pET-TEVR	GCAGCAGCCAACTCAGCTTCCTTTC
**Sequencing primers for plasmid pJC1**	
pJC1F	GACAGATTATCTGCAAACGGTGTGT
pJC1R	CAATGCTTAATCAGTGAGGCACCTA
**Oligonucleotides to determine the transcriptional start site of *gabT***	
GABTTS1R	GTTTTCGGCTCCGACCTGGGATTC
GABTTS2R	GTAAGACATTGGTGCACGGTAGAC
GABTTS3R	GTCCGTCCGTGGTACGCGTTGTC
**Oligonucleotides to generate DNA fragments for EMSAs (fragment names in bold letters)**	
**P*GabT***	
P_{GabT}F	ATTTCACGGGGGACTTCAAAGA
P_{GabT}R	GTATGAGAGATCTTCCACGGTTCCT
**FA1**	
P_{GabT}F	ATTTCACGGGGGACTTCAAAGA
GabrbsA1R	TAGGTGATGAGTATTCTCTCCGAGGC
**FA2**	
P_{GabT}F	ATTTCACGGGGGACTTCAAAGA
GabrbsA2R	GGCAACGAAGTTAATATGTCCATGAG
**FA3**	
P_{GabT}F	ATTTCACGGGGGACTTCAAAGA
GabrbsA3R	CATGAGGGCGAAGTTGTAGACAATA
**FA4**	
P_{GabT}F	ATTTCACGGGGGACTTCAAAGA
GabrbsA4R	CAATATTTCGCCCATATGGATAATT
**FA5**	
P_{GabT}F	ATTTCACGGGGGACTTCAAAGA
GabrbsA5R	TAATTGACAGGAGTTTAACGCCATG
**FA6**	
P_{GabT}F	ATTTCACGGGGGACTTCAAAGA
GabrbsA6R	CCATGGAAACCCCAACCCAAGACAT
**FB1**	
GabrbsB1F	ATGTCTTGGGTTGGGGTTTCCATGG
P_{GabT}R	GTATGAGAGATCTTCCACGGTTCCT
**FB2**	
GabrbsB2F	CATGGCGTTAAACTCCTGTCAATTA
P_{GabT}R	GTATGAGAGATCTTCCACGGTTCCT

Oligonucleotide	Sequence (5' → 3') and properties[a]
**FB3**	
GabrbsB3F	AATTATCCATATGGGCGAAATATTG
$P_{GabT}R$	GTATGAGAGATCTTCCACGGTTCCT
**FB4**	
GabrbsB4F	TATTGTCTACAACTTCGCCCTCATG
$P_{GabT}R$	GTATGAGAGATCTTCCACGGTTCCT
**FB5**	
GabrbsB5F	CTCATGGACATATTAACTTCGTTGCC
$P_{GabT}R$	GTATGAGAGATCTTCCACGGTTCCT
**FB6**	
GabrbsB6F	GTTGCCTCGGAGAGAATACTCATCAC
$P_{GabT}R$	GTATGAGAGATCTTCCACGGTTCCT
**FS**	
GabrbsB3F	AATTATCCATATGGGCGAAATATTG
GabrbsA2R	GGCAACGAAGTTAATATGTCCATGAG
**FS1**	
GabrbsB3F	AATTATCCATATGGGCGAAATATTG
GabrbsS1R	TTAATATGTCCATGAGGGCGAAGT
**FS2**	
GabrbsS2F	ATGGGCGAAATATTGTCTACAACTTC
GabrbsA2R	GGCAACGAAGTTAATATGTCCATGAG
**FS3**	
GabrbsS3F	CTGTCAATTATCCATATGGGCGAAATATTGT
GabrbsS3R	ACAATATTTCGCCCATATGGATAATTGACAG
**FS4**	
GabrbsS4F	CTACAACTTCGCCCTCATGGACATATTAACT
GabrbsS4R	AGTTAATATGTCCATGAGGGCGAAGTTGTAG
**FSM1**	
BSmutF	CATGATTTATATGGGCGAAATATTGTCTAC
GabrbsS1R	TTAATATGTCCATGAGGGCGAAG
**FSM2**	
GabrbsB3F	AATTATCCATATGGGCGAAATATTG
BSmutR	TTACTAGGTTTATGAGGGCGAAGTTGTAGAC
**FSM12**	
BSmutF	CATGATTTATATGGGCGAAATATTGTCTAC
BSmutR	TTACTAGGTTTATGAGGGCGAAGTTGTAGAC
**NC1**	
NCldhF	GTGGACAAAAACGTCAAGATTATGA
NCldhR	GAGCAGTGCAGAGACTGGGACTG
**NC2**	
NC55bpF	TAGACAACAGTTTGTATCTCACCTC
NC55bpR	GTATGAGAGATCTTCCACGGTTCCT
**NC3**	
NC31bpF	CACAGGAGGAACCGTGGAAGATCTCTCATAC
NC31bpR	GTATGAGAGATCTTCCACGGTTCCTCCTGTG
**NC4**	
NC76bpF	TCGGAGAGAATACTCATCACCTAGAC
NC76bpR	GTATGAGAGATCTTCCACGGTTCCT

[a]) Overlaps for Gibson assembly are written in bold letters. Restriction sites are underlined. Red letters indicate mutated bases in comparison to the template.

TABLE S2. Genes with at least 2-fold increased or decreased mRNA levels (p value ≤0.05) in WT cells grown in modified CGXII medium lacking $(NH_4)_2SO_4$ with either GABA or glucose as carbon sources. Genes with a more than 10-fold altered ratio are marked in grey.

**Locus tag**	**Gene name**	**Annotated function**	**mRNA ratio GABA/ Glucose**	**p value**
cg0052		siderophore ABC transporter, permease	2.05	0.040
cg0053		siderophore ABC transporter, ATPase	2.59	0.022
cg0078		put. membrane protein	4.65	0.000
cg0083		put. nicotinamide mononucleotide uptake permease, PnuC-family	10.08	0.000
cg0105		hypothetical protein	2.05	0.004
cg0111		hypothetical protein	3.82	0.001
cg0120		put. esterase/lipase/thioesterase family protein, hydrolase	2.38	0.000
cg0365		put. membrane protein	2.36	0.005
cg0387	*adhE*	mycothiol-dependent formaldehyde dehydrogenase	2.05	0.000
cg0518	*hemL*	glutamate-1-semialdehyde 2,1-aminomutase	2.15	0.008
cg0519		protein of histidine phosphatase superfamily, phosphoglycerate mutase-like protein	2.17	0.003
cg0520	*ccsX*	periplasmic thioredoxin	2.08	0.002
cg0522	*ccsA*	transmembrane disulfide interchange protein involved in cytochrome *c* biogenesis, DsbD family	2.38	0.001
cg0523		transmembrane cytochrome *c* biogenesis, ResB-family	2.34	0.002
cg0524	*ccsB*	transmembrane cytochrome *c* assembly protein, CcsA family	2.78	0.001
cg0565	*gabR*	put. transcriptional regulator, similar to PurR of *Bacillus subtilis* that regulates purine catabolism	4.28	0.004
cg0566	*gabT*	4-aminobutyrate aminotransferase, AT class II	86.90	0.001
cg0567	*gabD*	succinate semialdehyde dehydrogenase	77.87	0.001
cg0568	*gabP*	GABA-specific permease	64.83	0.001
cg0569		put. Cd^{2+}/cation-transporting P-type ATPase	2.35	0.008
cg0575		put. secreted protein	2.30	0.001
cg0637	*creC*	put. NAD^+-dependent 4-hydroxybenzaldehyd dehydrogenase subunit (EC 1.2.1.64)	2.74	0.009
cg0638	*creD*	put. *p*-cresol methylhydroxylase subunit	3.39	0.000
cg0639	*creE*	ferredoxin reductase	2.84	0.004
cg0640	*creF*	ferredoxin	2.88	0.003
cg0641	*creG*	put. 4-hydroxybenzyl-alcohol dehydrogenase	2.74	0.019
cg0642	*creH*	put. PEP-utilizing enzyme	2.69	0.017
cg0644	*creI*	put. pyruvate phosphate dikinase	3.19	0.040

**Locus tag**	**Gene name**	**Annotated function**	**mRNA ratio GABA/ Glucose**	**p value**
cg0701		put. drug/metabolite transporter, DMT superfamily	6.43	0.003
cg0776		put. ABC-type siderophore transporter, secreted substrate-binding lipoprotein	3.54	0.000
cg0796	*prpD1*	2-methylcitrate dehydratase	2.74	0.001
cg0797	*prpB1*	2-methylisocitrate lyase	5.22	0.000
cg0798	*prpC1*	2-methylcitrate synthase	3.48	0.000
cg0932		put. membrane protein	2.52	0.000
cg0968		put. ATP-dependent helicase	2.07	0.008
cg0997	*cgtS2*	two component histidine kinase	2.07	0.005
cg0998	*htrA*	secreted serine protease	2.53	0.001
cg1045		hypothetical protein, conserved	2.08	0.001
cg1069	*gapB*	glyceraldehyde 3-phosphate dehydrogenase involved in gluconeogenesis	2.09	0.000
cg1082		put. membrane protein	2.36	0.003
cg1083	*cgtS10*	two component histidine kinase	2.15	0.001
cg1084	*cgtR10*	two component response regulator	2.29	0.000
cg1087		put. membrane protein	2.26	0.002
cg1091		hypothetical protein	7.78	0.000
cg1095		hypothetical protein	2.02	0.011
cg1106		hypothetical protein, conserved	2.03	0.015
cg1145	*fumC*	fumarase	2.21	0.000
cg1179		put. sensory box/GGDEF-family membrane protein	2.34	0.002
cg1180		put. glycosyltransferase	2.16	0.001
cg1182		put. membrane protein	2.15	0.002
cg1201		hypothetical protein	2.08	0.006
cg1202		hypothetical protein, conserved	2.25	0.003
cg1271	*sigE*	ECF-type sigma factor	2.41	0.003
cg1292		put. flavin-containing monooxygenase	3.12	0.001
cg1293		put. secreted protein	2.03	0.000
cg1300	*cydB*	cytochrome *bd* oxidase, subunit II	2.04	0.001
cg1392		put. transcriptional regulator, CRO/CI-family, HTH_3-family	2.08	0.013
cg1410	*rbsR*	transcriptional repressor, LacI family	3.15	0.002
cg1411	*rbsA*	ribose/xylose ABC transporter, ATPase	3.01	0.005
cg1412	*rbsC*	ribose/xylose ABC transporter, permease	2.68	0.003
cg1413	*rbsB*	ribose/xylose ABC transporter, secreted sugar-binding protein	2.86	0.002
cg1447	*zrf*	zinc exporter, cation diffusion facilitator	2.21	0.001

Locus tag	Gene name	Annotated function	mRNA ratio GABA/ Glucose	p value
cg1471		hypothetical protein	2.29	0.043
cg1476	*thiC*	phosphomethylpyrimidine synthase	2.01	0.003
cg1498		put. RecG-like helicase	2.34	0.003
cg1513	*tnp23a*	transposase, put. pseudogene of CGP1 region	2.05	0.000
cg1514		put. secreted protein, CGP1 region	2.78	0.019
cg1515	*tnp24a*	transposase, put. pseudogene of CGP1 region	2.18	0.000
cg1516		hypothetical protein, CGP1 region	2.18	0.003
cg1517		put. secreted protein, CGP1 region	2.05	0.005
cg1543	*uriH*	inosine-uridine preferring nucleoside hydrolase	2.74	0.000
cg1545	*uriT*	permease of the major facilitator superfamily	2.59	0.003
cg1546	*rbsK1*	put. ribokinase protein	4.36	0.000
cg1547	*uriR*	transcriptional regulator of uridine utilization and ribose transport, LacI family	4.08	0.000
cg1617		put. GTP-binding protein EngA	2.29	0.001
cg1662		put. secreted protein	2.29	0.001
cg1673	*ppmN*	polyprenol-phosphate-mannose synthase domain 2	2.16	0.024
cg1683		put. superfamily II DNA and RNA helicase	2.93	0.009
cg1697	*aspA*	aspartate ammonia-lyase	2.50	0.000
cg1734	*hemH*	ferrochelatase	2.49	0.001
cg1759		put. Fe-S cluster assembly protein	2.17	0.004
cg1760	*sufU*	cysteine desulfhydrase	2.24	0.003
cg1761	*sufS*	Fe-S cluster assembly protein	2.08	0.008
cg1762	*sufC*	Fe-S cluster assembly ATPase	2.22	0.001
cg1763	*sufD*	Fe-S cluster assembly membrane protein	2.63	0.000
cg1764	*sufB*	Fe-S cluster assembly protein	2.57	0.004
cg1765	*sufR*	transcriptional repressor of the *suf* operon, ArsR-family	3.03	0.000
cg1895		put. secreted protein, CGP3 region	3.14	0.000
cg1901		hypothetical protein, CGP3 region	2.17	0.004
cg1902		put. secreted protein, CGP3 region	2.30	0.001
cg1903		put. ABC-type multidrug transport system, ATPase	2.35	0.000
cg1904		put. membrane protein, CGP3 region	2.48	0.001
cg1905		hypothetical protein, CGP3 region	2.78	0.001
cg1906		hypothetical protein, CGP3 region	3.06	0.000
cg1907		put. phosphopantothenoylcysteine synthetase/decarboxylase, CGP3 region	3.47	0.000
cg1908		hypothetical protein, CGP3 region	3.20	0.000
cg1942		put. secreted protein, CGP3 region	2.30	0.025
cg1943		hypothetical protein, CGP3 region	2.16	0.007

Locus tag	Gene name	Annotated function	mRNA ratio GABA/ Glucose	p value
cg1955		put. secreted protein, CGP3 region	2.11	0.000
cg1956	*recJ*	single-stranded-DNA-specific exonuclease	2.33	0.042
cg1961		hypothetical protein, CGP3 region	2.31	0.035
cg1963		put. superfamily II DNA/RNA helicase, CGP3 region	2.01	0.000
cg2004		protein similar to 232 protein-lactobacillus bacteriophage g1e	3.24	0.028
cg2046		hypothetical protein, CGP3 region	2.04	0.001
cg2051		hypothetical protein, CGP3 region	2.06	0.008
cg2052		put. secreted protein, CGP3 region	2.83	0.002
cg2068		hypothetical protein, CGP3 region	2.48	0.049
cg2069	*psp1*	put. secreted protein, CGP3 region	2.18	0.002
cg2094		hypothetical protein	2.04	0.001
cg2111	*hrpA*	put. ATP-dependent RNA helicase	2.31	0.000
cg2157	*terC*	transmembrane tellurium resistance protein	3.48	0.000
cg2176	*infB*	translation initiation factor IF-2, GTPase	2.08	0.008
cg2265	*smc*	chromosome segregation ATPase	2.17	0.001
cg2270		hypothetical protein, conserved	2.60	0.000
cg2271		put. secondary Co^{2+}/Zn^{2+}/Cd^{2+} efflux transporter, cation diffusion facilitator (CDF) family	2.24	0.003
cg2340		put. ABC-type amino acid transport system, secreted component	2.07	0.003
cg2422	*lipB*	lipoyltransferase	2.45	0.000
cg2430		hypothetical protein	4.01	0.000
cg2477		hypothetical protein, conserved	3.49	0.003
cg2555		hypothetical protein	2.86	0.003
cg2564		hypothetical protein	7.22	0.001
cg2572		hypothetical protein, conserved	3.43	0.000
cg2623	*pcaI*	β-ketoadipate succinyl-CoA transferase subunit	2.27	0.001
cg2624	*pcaR*	transcriptional repressor, IclR-family	4.32	0.000
cg2625	*pcaF*	β-ketoadipyl-CoA thiolase	2.98	0.003
cg2626	*pcaD*	β-ketoadipate enol-lactone hydrolase	2.43	0.002
cg2634	*catC*	muconolactone isomerase	2.82	0.000
cg2635	*catB*	chloromuconate cycloisomerase	2.48	0.001
cg2636	*catA1*	catechol 1,2-dioxygenase	4.52	0.001
cg2637	*benA*	benzoate 1,2-dioxygenase α subunit aromatic ring hydroxylation dioxygenase A	4.20	0.003
cg2638	*benB*	benzoate dioxygenase small subunit	4.14	0.001

Locus tag	Gene name	Annotated function	mRNA ratio GABA/ Glucose	p value
cg2639	*benC*	benzoate 1,2-dioxygenase ferredoxin reductase subunit	2.20	0.001
cg2640	*benD*	*cis*-diol dehydrogenase	2.18	0.004
cg2641	*benR*	transcriptional regulator, LuxR-family	2.65	0.006
cg2685		put. short chain dehydrogenase/reductase	2.50	0.002
cg2823		put. dehydrogenase or related protein	2.77	0.000
cg2828		put. membrane protein	2.13	0.003
cg2837	*sucC*	succinyl-CoA synthetase, β subunit, ADP-forming	2.05	0.005
cg2875		hypothetical protein, mycoloylated	2.32	0.040
cg2888	*phoR*	two component response regulator	2.12	0.001
cg2958	*butA*	L-2,3-butanediol dehydrogenase/acetoin reductase	2.40	0.001
cg2962		put. enzyme involved in biosynthesis of extracellular polysaccharides	2.57	0.005
cg3001	*cosR*	put. transcriptional regulator, MarR-family	2.34	0.001
cg3003	*cps*	non-ribosomal peptide synthetase	3.07	0.001
cg3045	*glnH*	secreted glutamate-binding lipoprotein	2.53	0.001
cg3046	*pknG*	serine/threonine protein kinase	2.36	0.007
cg3077		put. membrane protein	2.16	0.007
cg3114	*cysN*	sulfate adenyltransferase, subunit 1	2.67	0.001
cg3115	*cysD*	sulfate adenylyltransferase, subunit 2	2.88	0.002
cg3116	*cysH*	phosphoadenosine-phosphosulfate reductase	2.38	0.001
cg3117	*cysX*	ferredoxin-like protein	2.24	0.005
cg3161		put. membrane protein	2.25	0.005
cg3212		put. carboxymuconolactone decarboxylase subunit	4.50	0.000
cg3237	*sodA*	manganese superoxide dismutase	2.45	0.004
cg3280		put. secreted protein, horizontally transferred gene	2.54	0.003
cg3281	*copB*	Cu^{2+}/cation-transporting ATPase transmembrane protein	2.01	0.003
cg3282		put. Cu^{2+}/heavy metal binding transport protein,	2.28	0.012
cg3283		hypothetical protein	2.16	0.014
cg3327	*dps*	DNA-binding protein from starved cells, stores Fe^{3+}	2.71	0.006
cg3332	*qor3*	put. NADPH:quinone oxidoreductase	2.13	0.001
cg3350	*nagK*	fumarylpyruvate hydrolase	2.13	0.001
cg3367		put. ABC-type multidrug transport system, ATPase	2.12	0.004
cg3368		put. ABC-transporter, permease	2.47	0.003
cg0133	*abgT*	p-aminobenzoyl-glutamate transporter	0.39	0.008
cg0134	*abgB*	metal-dependent amidase/aminoacylase/ carboxypeptidase, AbgB homolog	0.46	0.008

Locus tag	Gene name	Annotated function	mRNA ratio GABA/ Glucose	p value
cg0158		put. transport protein, MFS family	0.49	0.008
cg0197	*iolC*	carbohydrate kinase, myo-inositol catabolism	0.42	0.002
cg0212		put. phosphate isomerase/epimerase, conserved	0.50	0.000
cg0277	*dccT*	dicarboxylate uptake system for succinate, fumarate or L-malate, DASS family	0.34	0.001
cg0286		put. membrane protein, conserved	0.36	0.005
cg0304		put. membrane protein	0.47	0.001
cg0404		put. protein of nitroreductase family, conserved	0.39	0.000
cg0411		put. membrane protein	0.49	0.002
cg0414	*wzz*	cell surface polysaccharide biosynthesis/chain length determinant protein	0.40	0.002
cg0506		ABC transporter for spermidine/putrescine/iron(III), ATPase	0.47	0.003
cg0507		ABC-transporter for spermidine/putrescine/iron(III), permease	0.37	0.004
cg0508		ABC-transporter for spermidine/putrescine/iron(III), secreted substrate-binding lipoprotein	0.28	0.001
cg0527	*glyR*	transcriptional regulator, ArsR family	0.50	0.050
cg0544		put. membrane protein	0.38	0.044
cg0545	*pitA*	low-affinity phosphate transport protein	0.22	0.008
cg0623	*cbrV*	put. cobalamin ECF transporter, transmembrane component	0.43	0.000
cg0624		put. secreted oxidoreductase	0.41	0.005
cg0665		put. serine protease	0.46	0.000
cg0772		put. sugar efflux permease, MFS type	0.49	0.005
cg0924		putative siderophore ABC transporter, secreted substrate-binding lipoprotein	0.46	0.002
cg0952	*mctB*	put. integral membrane protein	0.46	0.014
cg0953	*mctC*	monocarboxylic acid transporter	0.38	0.004
cg1001	*mscL*	large conductance mechanosensitive channel, MscL family	0.47	0.003
cg1214	*nadS*	cysteine desulfurase-like protein involved in Fe-S cluster assembly, required for maturation of NadA	0.46	0.001
cg1218	*ndnR*	transcriptional repressor, NrtR family	0.48	0.007
cg1290	*metE*	5-methyltetrahydropteroyltriglutamate-homocysteine methyltransferase	0.27	0.003
cg1347		put. secreted phospholipid phosphatase	0.40	0.000
cg1409	*pfkA*	6-phosphofructokinase	0.47	0.001

Locus tag	Gene name	Annotated function	mRNA ratio GABA/ Glucose	p value
cg1451	*serA*	phosphoglycerate dehydrogenase	0.32	0.006
cg1479	*malP*	maltodextrin phosphorylase	0.38	0.003
cg1482		put. Zn-dependent hydrolase, including glyoxylases	0.44	0.002
cg1487	*leuC*	isopropylmalate isomerase large subunit	0.33	0.001
cg1488	*leuD*	isopropylmalate isomerase small subunit	0.47	0.014
cg1492	*gpsA*	NADPH-dependent glycerol-3-phosphate dehydrogenase	0.33	0.023
cg1537	*ptsG*	glucose-specific EIIABC component of PEP-dependent phosphotransferase system (PTS)	0.41	0.000
cg1580	*argC*	N-acetyl-γ-glutamyl-phosphate reductase	0.47	0.012
cg1710	*uppP*	undecaprenyl pyrophosphate phosphatase	0.47	0.001
cg2181	*oppA*	peptide ABC-transporter, secreted component	0.37	0.007
cg2182	*oppB*	peptide ABC-transporter, permease	0.39	0.002
cg2221	*tsf*	elongation factor Ts	0.44	0.001
cg2312	*gip*	put. hydroxypyruvate isomerase	0.35	0.000
cg2391	*aroG*	phospho-2-dehydro-3-deoxyheptonate aldolase	0.47	0.013
cg2429	*glnA*	glutamine synthetase I	0.46	0.003
cg2470		put. branched-chain amino acid ABC transporter, secreted substrate-binding lipoprotein	0.48	0.001
cg2539	*ectP*	ectoine/proline/glycine betaine carrier, BCCT family	0.46	0.010
cg2610		put. dipeptide/oligopeptide/nickel ABC-transporter, secreted component	0.49	0.000
cg2777		put. membrane protein, conserved	0.47	0.002
cg2843	*pstB*	phosphate ABC-transporter, ATPase	0.43	0.011
cg2844	*pstA*	phosphate ABC-transporter, permease	0.32	0.000
cg2845	*pstC*	phosphate ABC-transporter, permease	0.16	0.006
cg2846	*pstS*	phosphate ABC-transporter, secreted phosphate-binding lipoprotein	0.17	0.003
cg2870	*dctA*	secondary H^+/Na^+:C_4-dicarboxylate symporter, dicarboxylate/amino acid:cation symporter (DAACS) family	0.41	0.029
cg2922		put. transcriptional regulator, IclR-family	0.47	0.000
cg2925	*ptsS*	sucrose-specific EIIABC component of PTS	0.41	0.000
cg2938	*siaF*	sialic acid ABC-transporter, permease	0.44	0.000
cg2939	*siaG*	sialic acid ABC-transporter, fused permease and ATPase components	0.35	0.001
cg2940	*siaI*	sialic acid ABC-transporter, duplicated ATPase domains	0.38	0.002
cg2964	*guaB1*	inosine-5-monophosphate dehydrogenase	0.43	0.002

**Locus tag**	**Gene name**	**Annotated function**	**mRNA ratio GABA/ Glucose**	**p value**
cg3011	*groEL*	chaperone GroEL	0.50	0.002
cg3054	*purT*	5-phosphoribosylglycinamide transformylase	0.42	0.005
cg3107	*adhA*	Zn-dependent alcohol dehydrogenase	0.33	0.004
cg3149	*alaT*	alanine aminotransferase	0.35	0.005
cg3219	*ldhA*	NAD-dependent L-lactate dehydrogenase	0.38	0.010
cg3226		L-lactate permease, MFS-type	0.12	0.007
cg3227	*lldD*	menaquinone-dependent L-lactate dehydrogenase	0.31	0.002
cg3277		hypothetical protein	0.50	0.001
cg3374	*cye1*	put. NADH-dependent flavin oxidoreductase, “old yellow enzyme” family	0.49	0.006
cg3382		put. dipeptide/tripeptide permease	0.46	0.001
cg3404		putative iron(III) dicitrate ABC transporter, substrate-binding lipoprotein	0.36	0.006

2 Supplementary Figures

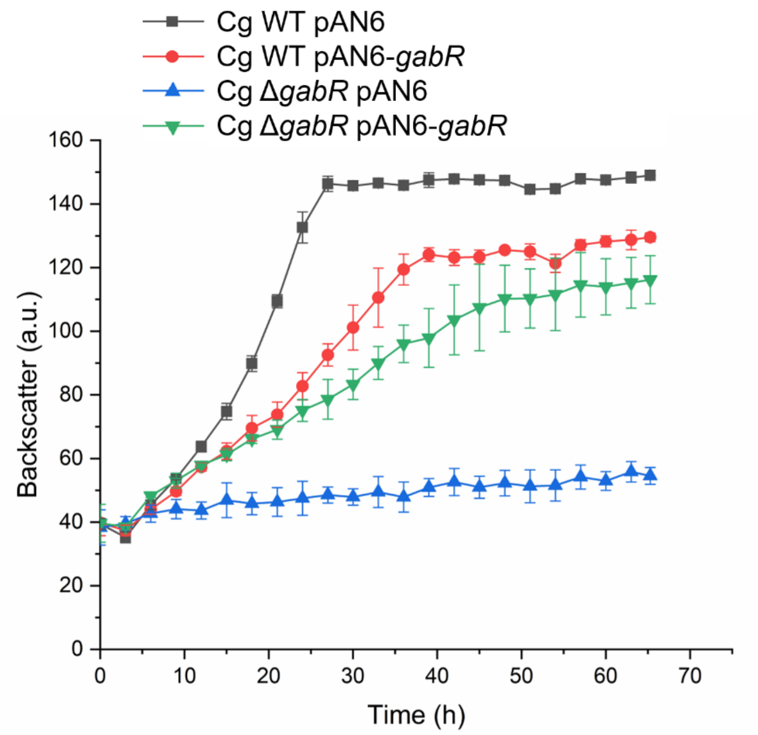

FIGURE S1. Influence of *gabR* overexpression on growth of *C. glutamicum* WT and the Δ*gabR* mutant with GABA as sole carbon and nitrogen source. Growth experiments were performed with a Biolector microcultivation system and FlowerPlates containing 750 µl CGXII minimal medium with 62.5 mM GABA and 50 µM IPTG for induction of *gabR* expression. BHIS medium was used for precultures and cells were washed with phosphate buffer before inoculation of the main cultures. Mean values and standard deviations of three biological replicates are shown.

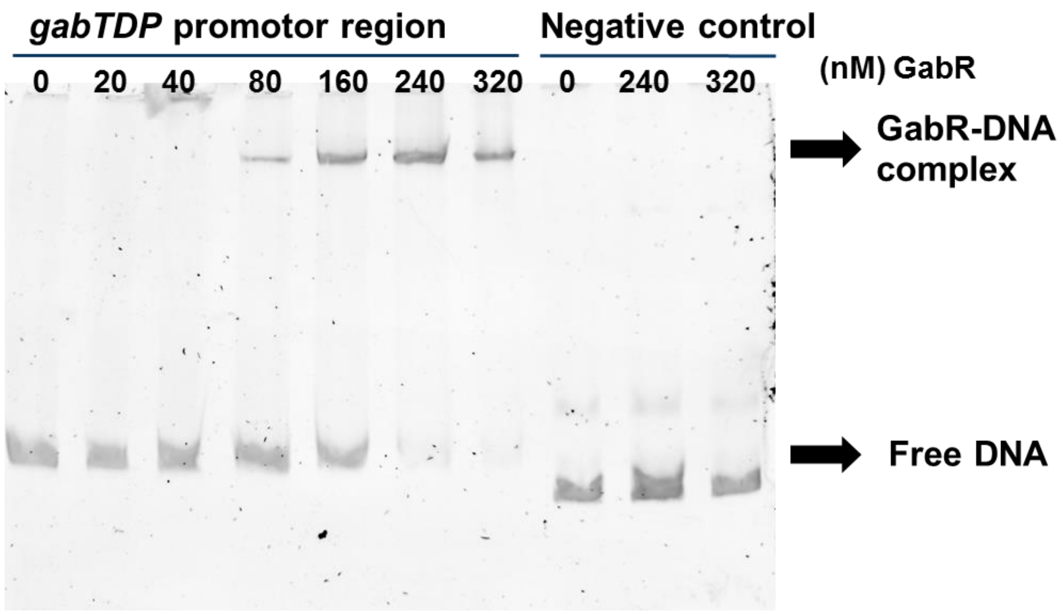

FIGURE S2. Electrophoretic mobility shift assays with purified GabR and the *gabTDP* promoter region. A 500 bp fragment covering the *gabT* promoter region (100 ng) was incubated with increasing concentrations (given in nM of monomers) of purified GabR as indicated. A 470 bp fragment of the *ldhA* gene of *C. glutamicum* was used as negative control. Electrophoresis was performed using 6% native polyacrylamide gels in an ice bath with TB buffer (89 mM Tris-HCl, 89 mM boric acid, pH 8.2) as running buffer (180 V, 45 minutes). Subsequently, the gel was stained with SYBR Green.

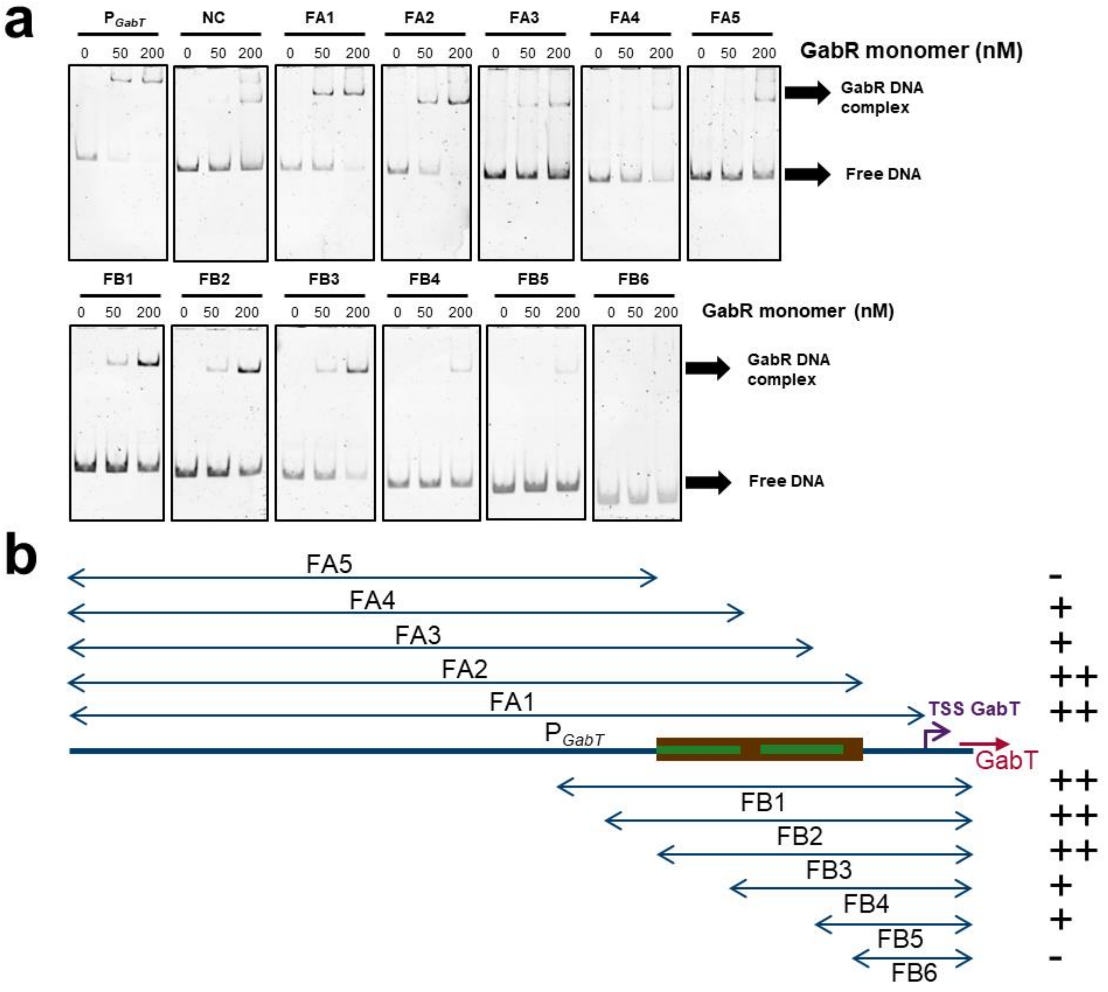

FIGURE S3. Localization of the GabR binding region within the *gabT* promoter. (a) EMSAs with different fragments of the *gabT* promoter region (100 ng) incubated with the indicated concentrations of purified GabR (given in nM monomeric GabR). A fragment of the *ldhA* gene of *C. glutamicum* was used as negative control. Electrophoresis was performed using 6% (w/v) native polyacrylamide gels in an ice bath with TB buffer (89 mM Tris-HCl, 89 mM boric acid, pH 8.2) as running buffer (180 V, 40 minutes). (b) Scheme showing the regions covered by the different fragments used in the EMSAs and the qualitative result of GabR binding to these fragments: ++, efficient binding, +, less efficient binding, -, no binding. The binding region derived from this experiment is marked in brown. Potential GabR binding sites are marked in green (compare Fig. 6). The translational start of *gabR* is marked with a red arrow, the TSS of *gabT* is marked with a purple arrow.

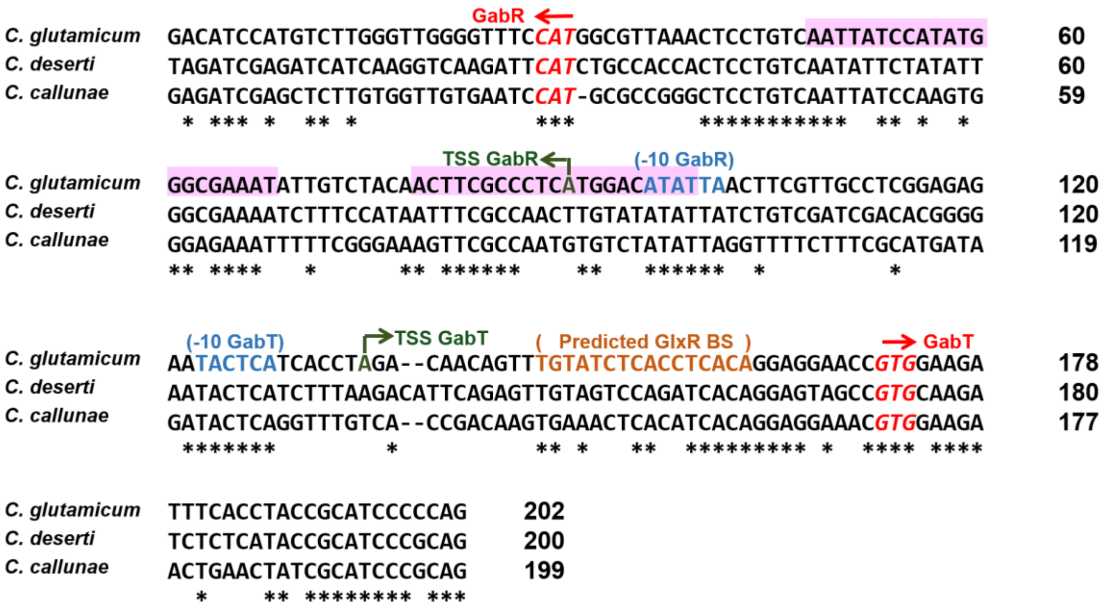

FIGURE S4. Alignment of the *gabR*-*gabT* intergenic regions of the indicated *Corynebacterium* species including 30 bp of each coding region. The start codons of *gabR* and *gabT* are marked in red, the TSSs of GabT and GabR are indicated with green arrows, and the predicted GlxR binding site is marked in brown. The GabR binding sites are marked with pink background.

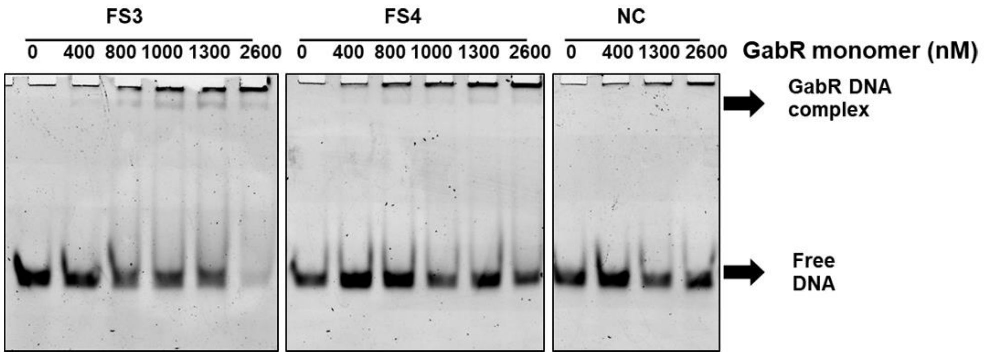

FIGURE S5. Electrophoretic mobility shift assays using purified GabR and 31 bp fragments of the *gabTDP* promoter region. Two 31 bp fragments (FS3 and FS4) covering one predicted GabR binding site each were generated by annealing of two complementary oligonucleotides and incubated with different concentrations of GabR (nM of a monomeric form) as indicated. As negative control, a 31 bp fragment of the downstream of the predicted binding sites was used. Electrophoresis was performed using 8% (w/v) native polyacrylamide gels in an ice bath with TB buffer (89 mM Tris-HCl, 89 mM boric acid, pH 8.2) as running buffer (180 V, 40 minutes).

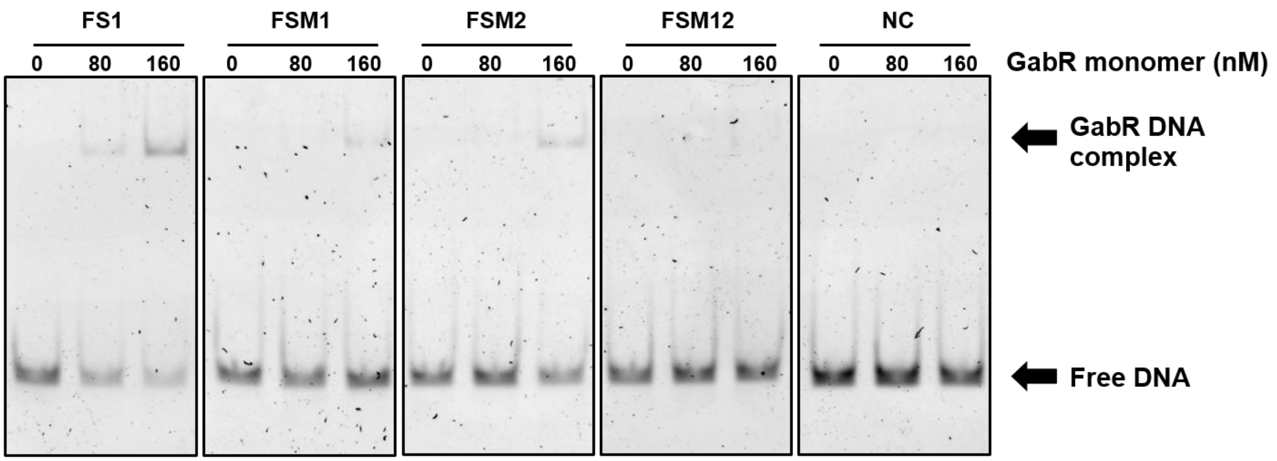

Binding site 1	Binding site 2	Fragment
...AATTATCCATATGGGCGAAAT...	ACTTCGCCCTCATGGACATAT...	FS1
...**C**AT**G**AT**TT**ATATGGGCGAAAT...	ACTTCGCCCTCATGGACATAT...	FSM1
...AATTATCCATATGGGCGAAAT...	ACTTCGCCCTCAT**AA**AC**C**TA**G**...	FSM2
...**C**AT**G**AT**TT**ATATGGGCGAAAT...	ACTTCGCCCTCAT**AA**AC**C**TA**G**...	FSM12

FIGURE S6. Mutation analysis of the two GabR binding sites in the *gabTDP* promoter region using electrophoretic mobility shift assays. The fragments (100 ng) were incubated with different GabR concentrations (given in nM of GabR monomer) as indicated. Fragments: FS1, 55 bp native *gabTDP* promoter region with both binding sites (positive control); FSM1, FS1 with four bps of the first binding site mutated (see scheme below the EMSA); FSM2, FS1 with four bps of the second binding site mutated; FSM12: FS1 with four bps of both binding sites mutated. The mutations were introduced using specific oligonucleotides as given in Table S1. A 55 bp fragment downstream of the predicted binding sites was used as negative control (NC). Electrophoresis was performed using 6% (w/v) native polyacrylamide gels in an ice bath with TB buffer (89 mM Tris-HCl, 89 mM boric acid, pH 8.2) as running buffer (180 V, 40 minutes).

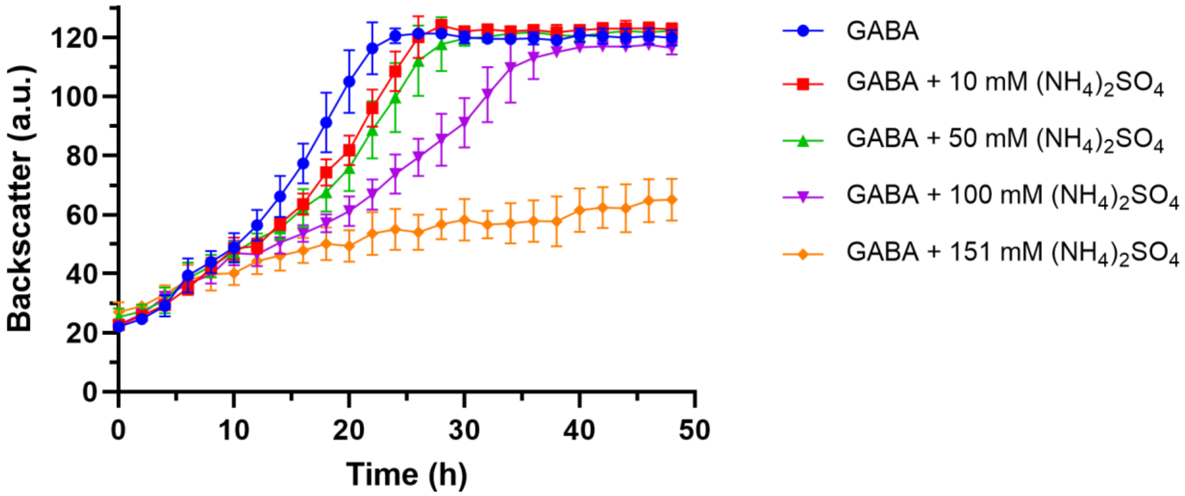

FIGURE S7. Influence of increasing $(NH_4)_2SO_4$ concentrations on growth of *C. glutamicum* WT with GABA as sole carbon source. Growth experiments were performed with a Biolector microcultivation system and FlowerPlates containing 750 µl CGXII minimal medium with 62.5 mM GABA. BHIS medium was used for precultures and cells were washed with phosphate buffer before inoculation of the main cultures. Mean values and standard deviations of three biological replicates are shown.

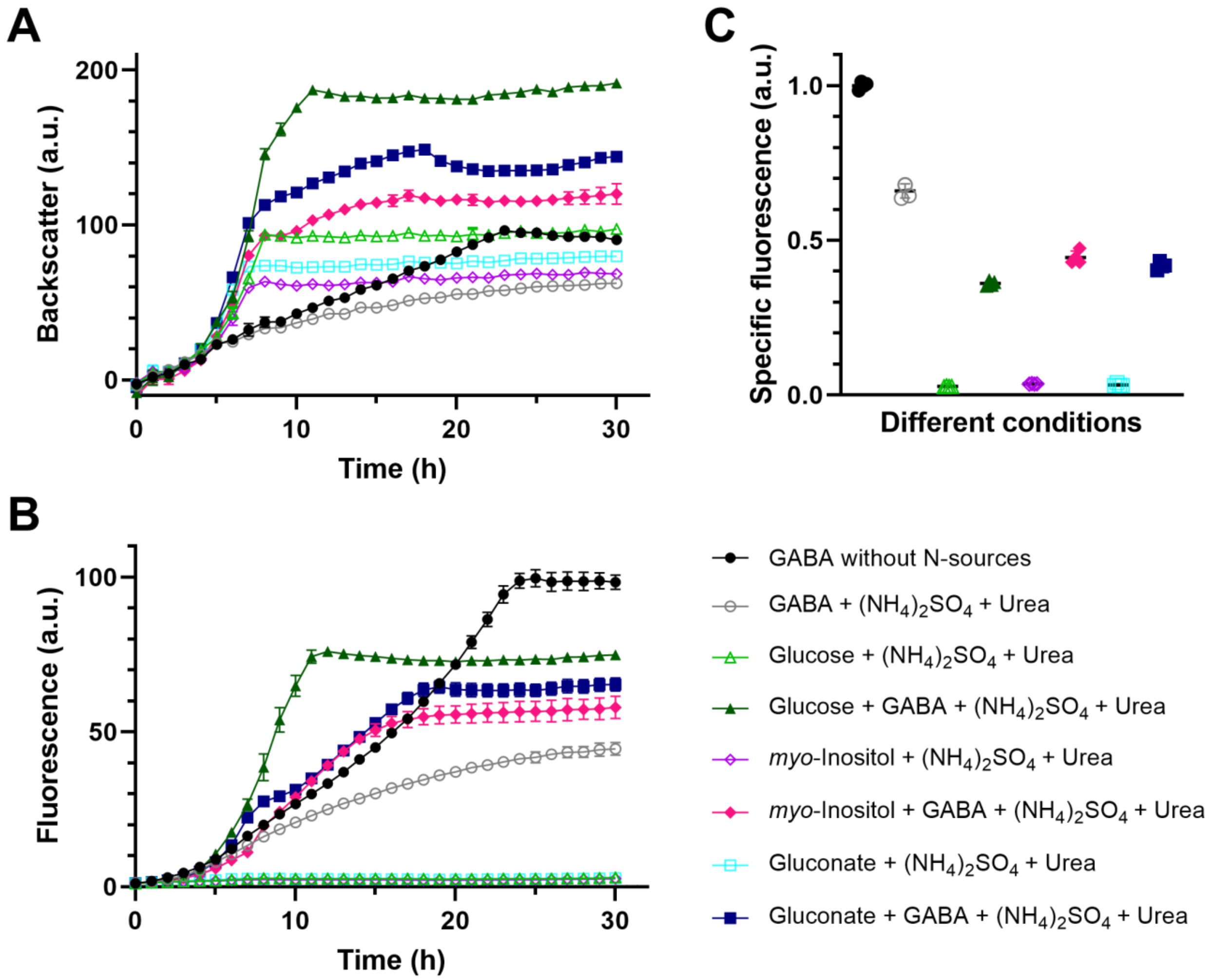

FIGURE S8. Promoter activity assay with glucose, *myo*-inositol, and gluconate. *C. glutamicum* WT cells transformed with the reporter plasmid pJC1-P$_{gabT}$-eYFP were precultivated in BHIS medium and washed with phosphate buffer before inoculation of the main cultures. The cultivations were performed as described in a Biolector microcultivation system at 30 °C and 1200 rpm using 750 µl CGXII minimal medium supplemented with GABA (62.5 mM), glucose (41.7 mM), *myo*-inositol (41.7 mM), gluconate (41.7 mM), $(NH_4)_2SO_4$ (151 mM), and urea (83 mM) as indicated. (a, b) Growth and eYFP fluorescence representing the activity of the *gabTDP* promoter of *C. glutamicum* WT with pJC1-P$_{gabT}$-eYFP. (c) Specific fluorescence (ratio fluorescence/backscatter) after 30 h of the cultures shown in (a) and (b). Mean values and standard deviations of three biological replicates are shown.

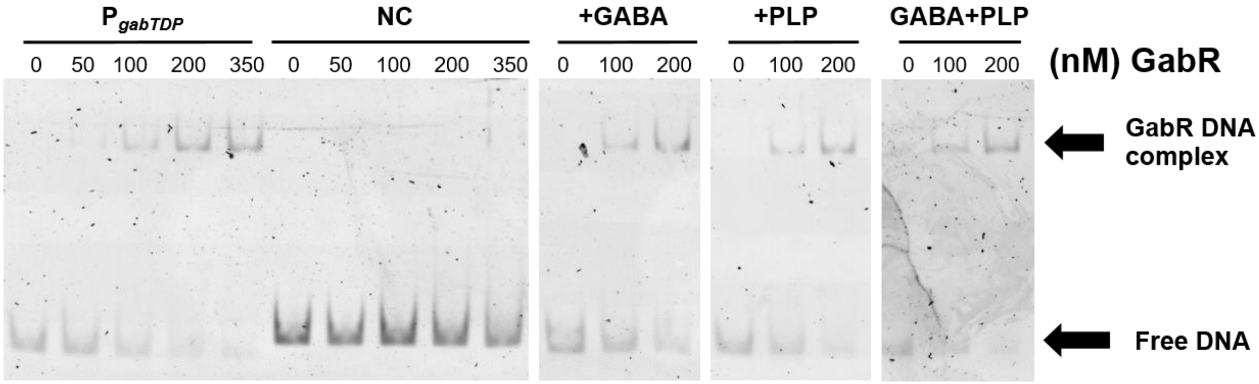

FIGURE S9. Influence of GABA and PLP on binding of GabR to the *gabTDP* promoter region. A 65 bp fragment of the *gabT* promoter region (100 ng) was incubated with increasing concentrations of purified GabR (nM of monomeric GabR) as indicated. A 76 bp fragment downstream of the predicted binding sites was used as negative control (NC).Electrophoresis was performed using 6% native polyacrylamide gels in an ice bath with TB buffer (89 mM Tris-HCl, 89 mM boric acid, pH 8.2) as running buffer (180 V, 40 minutes). GABA (5 mM) and PLP (0.05 mM) were added to the binding buffer.

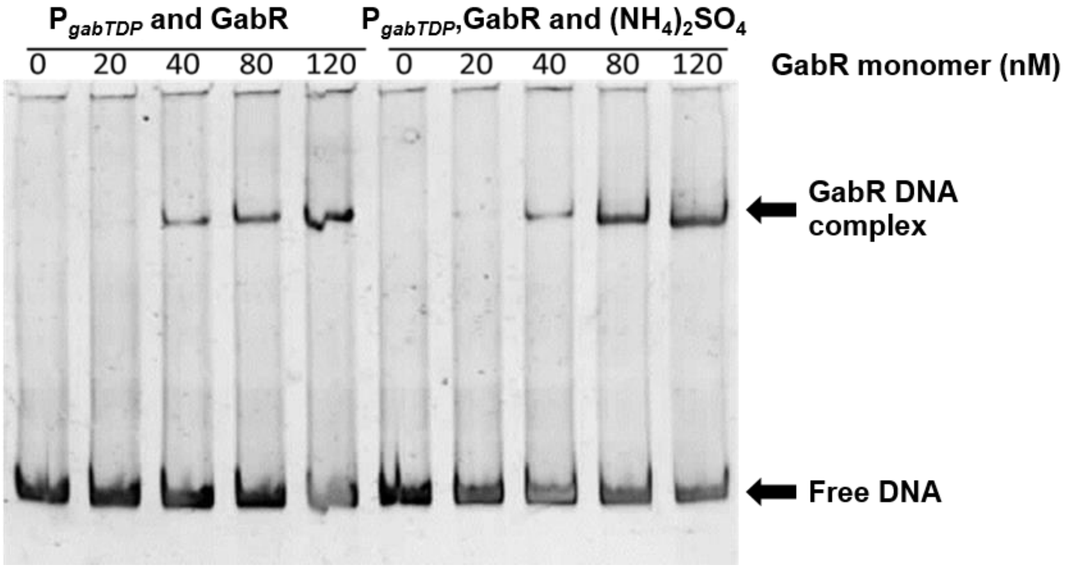

FIGURE S10. Influence of 50 mM ammonium sulfate on the binding of GabR to the *gabTDP* promoter region. A 500 bp fragment covering the *gabT* promoter region (300 ng) was incubated with increasing concentrations of purified GabR (nM of monomeric form) as indicated. Electrophoresis was performed using 6% native polyacrylamide gels in an ice bath with TB buffer (89 mM Tris-HCl, 89 mM boric acid, pH 8.2) as running buffer (180 V, 45 minutes).
